# Computational Study on the Influence of Mo/V Centers on the Electronic Structure and Hydrazine Reduction Capability of [MFe_3_S_4_]^3+/2+^ Complexes

**DOI:** 10.1021/acs.inorgchem.3c02072

**Published:** 2023-09-27

**Authors:** Maxim Barchenko, Thomas Malcomson, Sam P. de Visser, Patrick J. O’Malley

**Affiliations:** † Department of Chemistry, School of Natural Sciences, The University of Manchester, Manchester M13 9PL, U.K.; ‡ Manchester Institute of Biotechnology, The University of Manchester, 131 Princess Street, Manchester M1 7DN, U.K.; § Department of Chemical Engineering, The University of Manchester, Oxford Road, Manchester M13 9PL, U.K.

## Abstract

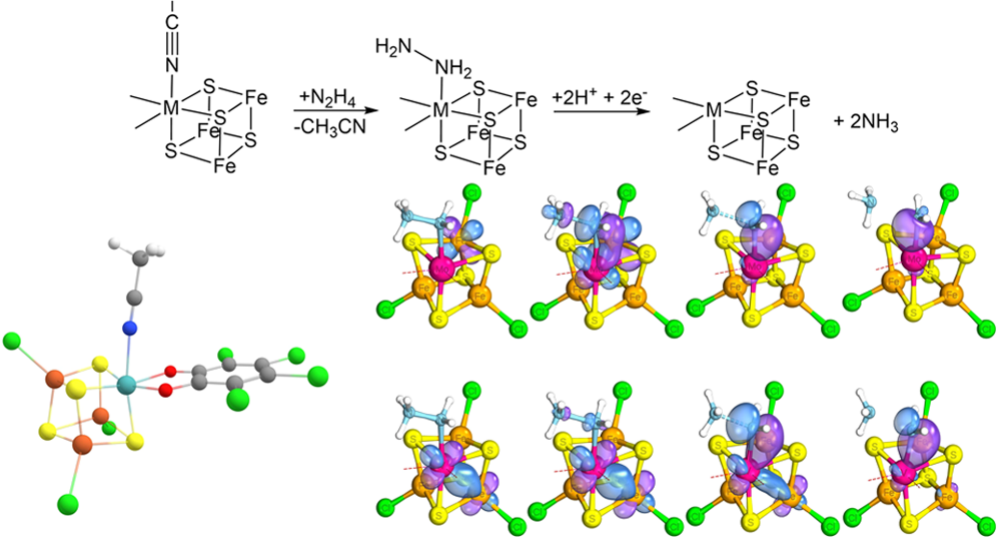

[MFe_3_S_4_] cubanes have for some time been of interest for their ability to mimic the electronic and geometric structure of the active site of nitrogenase, the enzyme responsible for fixing N_2_ to NH_3_. Nitrogenase naturally occurs in three forms, with the major difference being that the metal ion present in the cofactor active site is either molybdenum (FeMoco), vanadium (FeVco), or iron. The molybdenum and vanadium versions of these cofactors are more closely studied, owing to their larger abundance and rate of catalysis. In this study, we compare free energy profiles and electronic properties of the Mo/V cubanes at various stages during the reduction of N_2_H_4_ to NH_3_. Our findings highlight the differences in how the complexes facilitate the reaction, in particular, vanadium’s comparatively weaker ability to interact with the Fe/S network and stabilize reducing electrons prior to N–N bond cleavage, which may have implications when considering the lower efficiency of the vanadium-dependent nitrogenase.

## Abstract 2

In this work, we take an in-depth look at the hydrazine reduction reaction as catalyzed by [MoFe_3_S_4_] and [VFe_3_S_4_] complexes, focusing on the reaction energetics and fine details of electron movement between the intermediates. The key similarities and differences observed in the course of this investigation further elucidate the role vanadium and molybdenum centers play in facilitating this reaction, with implications for the nitrogenase enzymes.

## 1 Introduction

**Figure 1:**
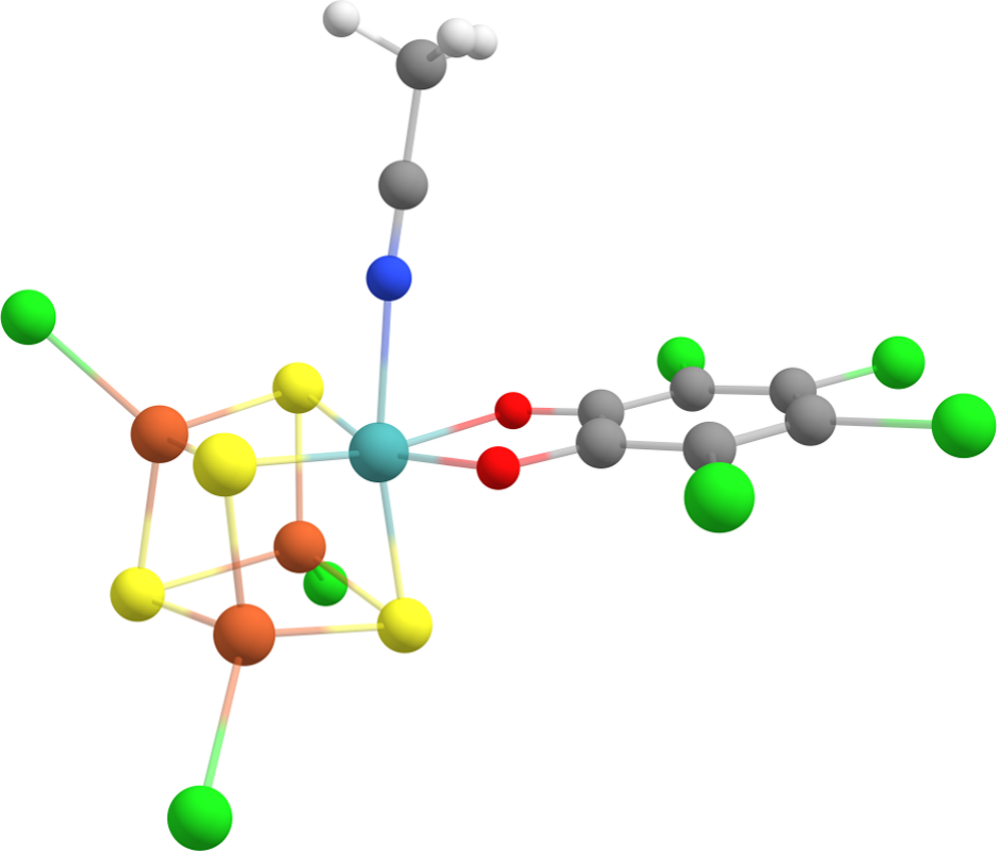
Structure of the [MoFe_3_S_4_]^3+^ cubane with a bound acetonitrile ligand. Color scheme: turquoise = Mo, yellow = S, dark orange = Fe, green = Cl, red = O, gray = C, and blue = N. The vanadium version differs only in Mo/V.

The nitrogenase enzymes are responsible for the biological conversion of atmospheric dinitrogen (N_2_) to ammonia (NH_3_). There are three main types of nitrogenases found in nature—the molybdenum-dependent,^1^ the vanadium-dependent,^2^ and the iron-only,^3^ utilizing the respective metal ions in the Fe/S frameworks of their cofactors, such as the Iron–Molybdenum cofactor (FeMoco), which are believed to be where the binding and reduction of N_2_ takes place.^4^ The Mo-dependent nitrogenase is the faster-acting of the three,^5^ with the V-dependent nitrogenase typically only expressed in Mo-deficient conditions, and the iron-only nitrogenase only in Mo/V deficient conditions.^6^ Owing to its earlier discovery and better efficiency, there have been significantly more studies on the Mo-dependent nitrogenase, and consequently, much more is known about it. Despite that, even for the Mo-dependent nitrogenase, many aspects of the mechanism by which it is able to bind and reduce dinitrogen remain uncertain (Figure 1).^7,8^

**Figure 2:**
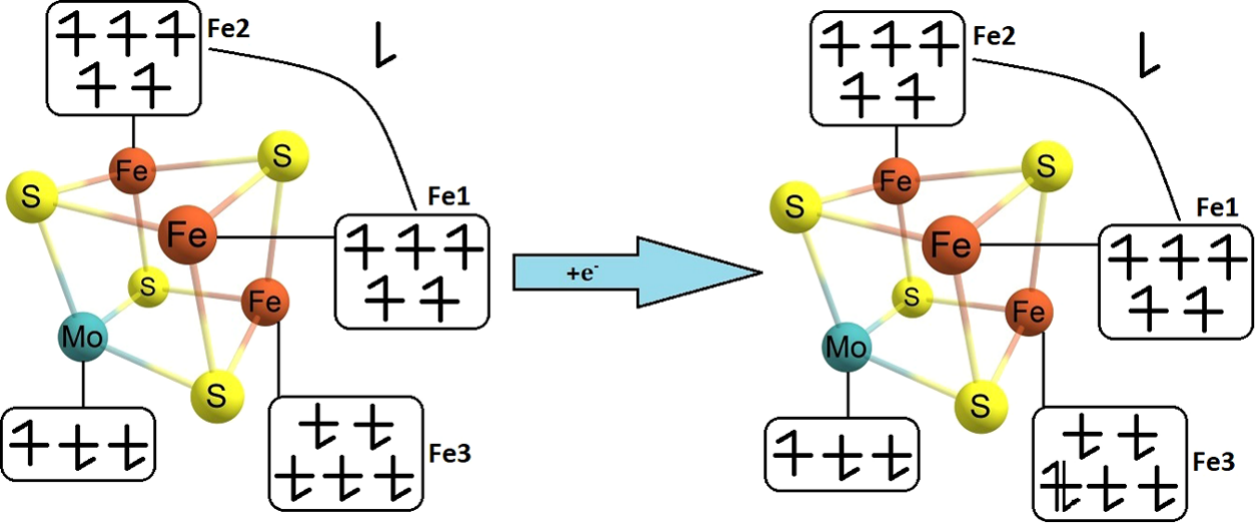
Electron configuration of the studied [MoFe_3_S_4_]^3+^ complex at its resting state (left) and after one electron reduction (right).

Early studies showed that [MoFe_3_S_4_]^3+^ cubanes can be utilized as promising model compounds for studying the properties and activity of nitrogenases’ FeMoco.^9,10^ They are experimentally known to catalyze the reduction of some of the substrates catalyzed by nitrogenase,^11,12^ notably hydrazine (N_2_H_4_), one of the key intermediates in the reduction of dinitrogen to ammonia.^13^ If FeMoco’s central structure is best described as two cubanes bridged by sulfurs, then the [MoFe_3_S_4_]^3+^ is effectively half of that, incorporating the Mo-containing cubane into its structure and with the interstitial carbide modeled by a sulfur. The complex is known to resemble the appropriate part of FeMoco’s crystallographic structure quite well, in addition to sharing certain key features of its electronic structure, such as mixed-valence Fe centers,^14^ non-Hund’s Mo electron configuration^15,16^ (Figure 2), and a ground-state spin of 3/2.^17,18^ These features make the complex a potentially viable model for FeMoco in terms of structure and functionality at a fraction of the computational cost. Furthermore, experimental studies performed to compare the relative hydrazine reduction activities of these molybdenum and vanadium cubanes suggested lower activity for the vanadium counterparts,^19,20^ as observed with the nitrogenases. Within this model, investigating the difference between Mo/V centers in the context of this reaction may therefore be useful in gaining insight into the role they play in nitrogenase. However, unlike FeMoco, in FeVco, the ground spin state and oxidation states of the metal ions have some debate surrounding them. While current thinking tends to suggest that the FeVco shares the same electronic structure and ground state as FeMoco (*S* = 3/2) with valencies [V^3+^, 3Fe^3+^, 4Fe^2+^],^6^ it should be noted that a recently published paper has proposed an alternate configuration, with an integer ground spin state and valencies [V^3+^, 4Fe^3+^, 3Fe^2+^]^21^ based on new EPR evidence. Therefore, for the sake of completeness and to account for both possibilities, we have included [VFe_3_S_4_]^3+^ and [VFe_3_S_4_]^2+^ complexes in the comparison with [MoFe_3_S_4_]^3+^. The vanadium complexes are distinguished in the text as isocharged and isoelectronic, relative to the Mo complex, respectively.

## 2 Methods

The computational software package ORCA^22^ version 4.2.1 was used for all calculations. Density functional theory (DFT) calculations were done using the BP86^23^ functional with D3BJ^24,25^ dispersion correction. The Ahlrichs def2-TZVP^26^ basis set was used on all atoms. The functional choice for nitrogenase and nitrogenase-like structures has been studied quite thoroughly. For geometry optimization, it is important to use sub-10% Hartree–Fock exchange functionals, as larger hybrid functionals result in unacceptably large errors in predicted distances for Fe–Fe and Fe–S interactions, among other issues.^27−29^ Further calculations with the TPSSh functional^30^ were performed in the initial benchmarking stage, resulting in comparable results both in terms of relative energies for energy profiles and geometries.

Broken-symmetry solutions were found by first converging to a high spin solution of the cluster (for [1], Ms = 8.5, 8.0, 7.5 for Mo, V(3+), and V(2+) complexes, respectively) and then selectively flipping the spin on iron atoms and optimizing on the broken-symmetry potential energy surface (for [1], Ms = 1.5, 1.0, 1.5 for Mo, V(3+), and V(2+) complexes respectively).

The conductor-like polarizible continuum model (CPCM) with the SMD model^31^ was used to implicitly describe solvation in acetonitrile. Adding solvation to calculations with these complexes is essential, as it both allows better simulation of the experimental reaction conditions and is vital for the correct geometry optimization of the complexes, particularly the charged intermediates. For instance, attempting to optimize a structure for the M-N_2_H_5_^+^ intermediate without accounting for solvation results in spontaneous dissociation of the proton from the complex. Vibrational frequency analysis was performed on all structures in order to confirm the ground and transition states as well as to calculate Gibbs free energies for the reaction free energy profiles. Reduction and protonation steps on the free energy profiles were calculated relative to the redox energy of cobaltocene and the deprotonation energy of lutidinium acid, respectively, which were the experimentally used reducing agent and proton donor in the referenced material.

IBOView^32^ software was used to localize the molecular orbitals produced by ORCA with the BP86 functional and view the resulting intrinsic bond orbitals (IBOs). IBOs provide the means to view MOs as more traditional bonding orbitals, which enables us to more easily visualize and track the flow of electrons as the reaction progresses.

Mössbauer parameters were calculated utilizing ORCA’s EPRNMR module to calculate the ρ and Δ*E*_Q_ values for each iron center and then manually calculating the shift δ with
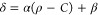
where α, *C*, and β are constants derived from calibration for the utilized functional and basis set, in this case being BP86 and def2-TZVP, respectively.^33^

## 3 Results and Discussion

### 3.1 Geometry and Electronic Structure

The complexes studied in this work exhibit a significant degree of antiferromagnetic coupling due to their geometry and electronic structure, which, in turn, stabilizes the complex and heavily influences its catalysis. Attempting to optimize the structure of the molybdenum complex on the high spin (*M*_S_ = 8.5) potential energy surface, for example, results in a structure 80 kcal mol^–1^ higher in energy than the broken-symmetry equivalent, and a geometry that does not resemble the experimental crystal structure, with longer metal–metal distances and a more regular cube-like shape of the cubane. IBO analysis of this high spin state shows no meaningful interaction between the Mo and Fe centers, with all Fe electrons strongly localized having a mixed valence of 2.33, and the Mo d-electrons either strongly localized or used in π-back-bonding to the acetonitrile ligand. Like in other works involving the same molybdenum complex,^34^ its ground state was determined to be the broken-symmetry *M*_S_ = 3/2 state, as shown in Figure 2, via analysis of J-coupling constants. We note that while our work utilizes the ground spin state Ms = 3/2 as determined experimentally for the isoelectronic Mo/V complexes, there is experimental data suggesting the [VFe_3_S_4_]^3+^ complex’s ground spin state is Ms = 0.^35^ Our calculations indicate that the Ms = 1 broken-symmetry solution is most energetically favorable by at least 8 kcal mol^–1^. Furthermore, the experimental data in question is obtained at temperatures close to 0 K and shows rapid population of Ms > 0 states at temperatures as low as 150 K, demonstrating the close-lying nature of these states. Given this and known complications in determining ground spin states for iron–sulfur clusters due to effects such as spin canting,^36^ our method utilizing BS-DFT may not provide a fully accurate description of the ground spin state of [VFe_3_S_4_]^3+^, which would require a multiconfigurational approach. For the purposes of this study, particularly taking into account that the reaction takes place under ambient conditions, whereby the population of the Ms = 0 state and the relative importance of the effect(s) that cause it can be reasonably assumed to diminish, we chose to utilize the lowest energy calculated ground spin state (in the starting structure [1]) of Ms = 1 for the [VFe_3_S_4_]^3+^ complex as a reasonable approximation, which provides an additional benefit of a more direct point of comparison to the other two structures.

**Figure 3:**
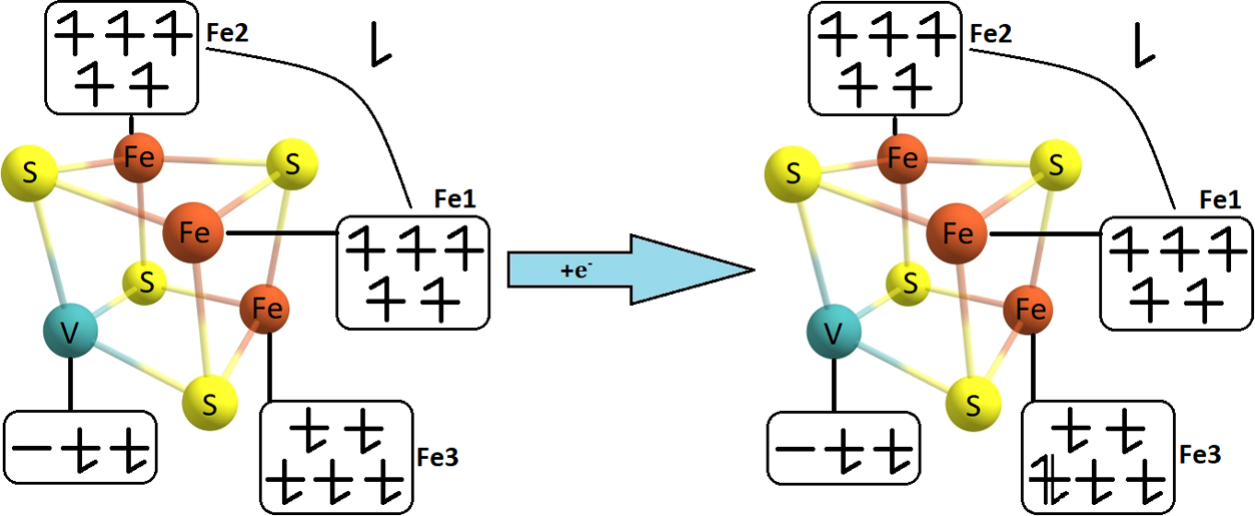
Electron configuration of the studied [VFe_3_S_4_]^3+^ (left) and [VFe_3_S_4_]^2+^ (right) complexes.

Test calculations on M-N_2_H_4_ and M-ACN complexes showed that there was no large preference between which of the 3 Fe centers were flipped—in all cases, the energetic difference between the broken-symmetry states was <1 kcal mol^–1^. For consistency, all energies presented here were calculated from the same broken-symmetry state (as depicted in Figures 2 and 3, with Fe3 flipped).

For all of the structures involved in the reaction pathways investigated, the spin and charge population analyses were closely monitored as part of verifying convergence to the correct electronic structure and to complement the data subsequently obtained from IBOs. On the whole, the data support the observations from the IBOs and further illustrate both the relative degree and importance of electron delocalization across the metal centers in each complex. The most notable example of electron delocalization in the system is between the Mo and Fe centers. By adopting an unusual non-Hund’s electron configuration with three unpaired electrons, one α, and two β, the antiferromagnetic coupling interaction is optimized toward the two α iron centers and the one “flipped” β iron center. The strength of this interaction effectively pulls the irons closer to the molybdenum, causing the distorted shape of the cubane, which is not observed in the high spin complex. This effect and interaction are observed in the vanadium complexes as well, albeit not quite to the same extent. A more detailed comparison between the high spin and broken-symmetry optimized geometries can be found in the Supporting Information (Table S1). The two α irons equally share one additional β electron between them. Further details of the electronic structure and notable differences between the molybdenum and vanadium complexes are discussed in the IBO section. Tables with Mulliken spin and charge population analyses are available in the Supporting Information for all calculated structures shown in the free energy profiles.

The Mössbauer parameters calculated for iron centers of the optimized Mo–N_2_H_4_ structure (Table 1) agree well with the experimental [MoFe_3_S_4_]^3+^ parameters,^37^ further supporting the capability of the chosen method to adequately describe the electronic structure of the important iron centers in the complex.

**Table 1:** Calculated Mössbauer Parameters of Mo–N_2_H_4_ Complex Relative to Experimentally Determined Parameters of [(Tp)MoFe_3_S_4_Cl_3_]^−^^37^

center	δ	Δ*E*_Q_	δ (exp.)	Δ*E*_Q_ (exp.)
Fe1	0.50	1.07	0.51	1.09
Fe2	0.50	1.08	0.51	1.09
Fe3	0.42	0.58	0.46	0.61

### 3.2 Reaction Free Energy Profiles

**Figure 4:**
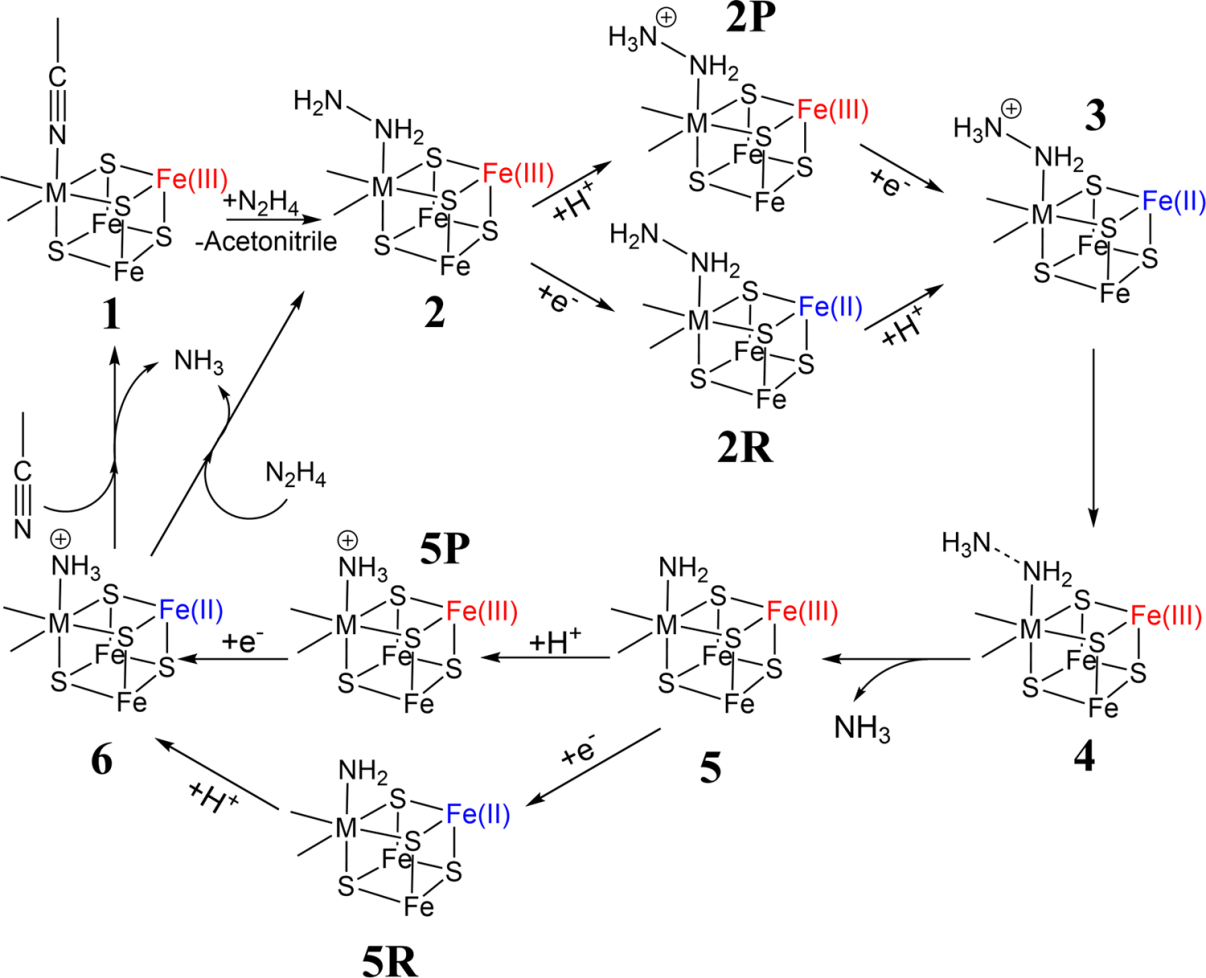
Reaction Scheme showing the hydrazine reduction mechanism investigated in this work. Parts of the structure have been omitted for clarity.

**Figure 5:**
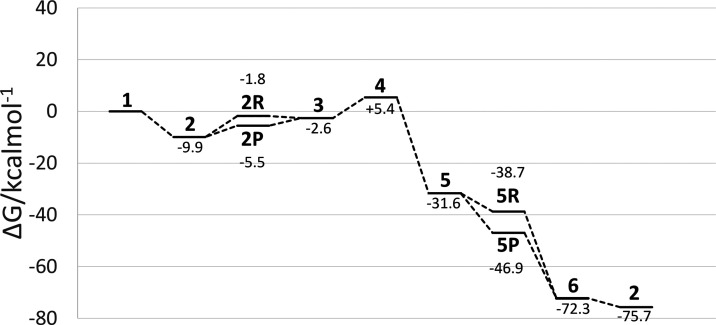
Free energy profile for the reduction of hydrazine with [MoFe_3_S_4_]^3+^.

The investigated reaction mechanism (Figure 4) begins when hydrazine binds to the initial complex structure by substituting the labile acetonitrile ligand. The hydrazine then undergoes a protonation and reduction step before arriving at the transition state, where N–N bond cleavage occurs, and the first NH_3_ molecule is liberated. The protonation and reduction steps occur once more, with the reaction terminating once the second NH_3_ molecule is substituted with hydrazine or acetonitrile. The energy profile (Figure 5) for this reaction is in fairly good agreement with the work done by Thorhallsson and Bjornsson.^34^ The reduction of N_2_H_4_ with the molybdenum complex proceeds with only a slight barrier to the first protonation/reduction step, with a slight preference of 3.7 kcal mol^–1^ for the protonation. The subsequent step is either a negligible barrier or downhill, followed by the transition-state barrier of 8.0 kcal mol^–1^, after which the reaction should readily follow a series of favorable steps to completion, with a preference of 8.2 kcal mol^–1^ for the reduction step over protonation first.

**Figure 6:**
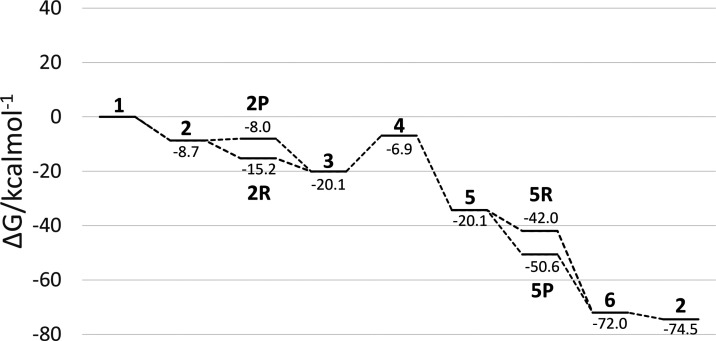
Free energy profile for the reduction of hydrazine with [VFe_3_S_4_]^3+^.

The energy profile of the isocharged [VFe_3_S_4_]^3+^ (Figure 6) complex is quite similar qualitatively to that of the molybdenum complex, especially after the N–N bond cleavage. Before the transition state, reduction is preferable as the first step by 7.2 kcal mol^–1^ over protonation, whereas after the transition state, protonation is instead preferable as the first step by 8.6 kcal mol^–1^. Overall, the isocharged vanadium energy pathway enjoys multiple consecutive exergonic steps before the N–N cleavage, whereas the molybdenum complex must overcome some minor energy barriers, albeit it ends up at a larger transition-state barrier of 13.2 kcal mol^–1^.

**Figure 7:**
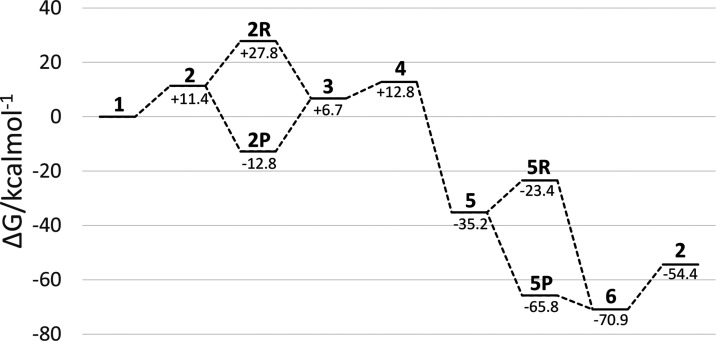
Free energy profile for the reduction of hydrazine with [VFe_3_S_4_]^2+^.

There is quite a stark difference when the energy profile of the isoelectronic vanadium complex [VFe_3_S_4_]^2+^ (Figure 7) is compared to those of the other two. Immediately substituting acetonitrile for hydrazine is not favorable, with an 11.4 kcal mol^–1^ barrier. Analysis of our data suggests that the hydrazine donates a relatively larger amount of electron density to the complex compared to the acetonitrile solvent; in the case of the isoelectronic vanadium complex, this results in significant disruption of the Fe–V interactions within the complex. For molybdenum and isocharge vanadium complexes, the Fe–M interactions are maintained at a similar level after substitution. This is likely the reason for such a large disparity in the acetonitrile substitution energies between the complexes and is further discussed in the IBO section.

Furthermore, isoelectronic vanadium’s energy profile shows a clear and significant preference for protonation over reduction in both steps, by 40.6 kcal mol^–1^ in the first step and by 42.4 kcal mol^–1^ in the second step, with an energy barrier for reduction of 19.5 kcal mol^–1^ after first protonation, and a transition-state barrier of only 6.1 kcal mol^–1^ by comparison. Finally, similar to the unfavorable substitution of acetonitrile for hydrazine, it is not favorable to substitute NH_3_ for hydrazine to restart the cycle, with a barrier of 16.5 kcal mol^–1^. Realistically, it would proceed by substitution for the abundant solvent at a lower barrier of 5.2 kcal mol^–1^ first. Overall, the energy profiles alone give an indication that the isoelectronic vanadium complex would be the least effective of the three at catalysis under the conditions employed, with energy barriers suggesting this is due to poor stabilization of reducing equivalents. The resemblance of the isocharged complexes’ energy profiles in contrast to the disparity between the isoelectronic ones gives some credence to the alternate spin state^21^ as plausible if it is meant to fulfill the same role as the molybdenum equivalent.

### 3.3 IBOs and Electron Flow

Analysis of the bonding orbitals of the different complexes over the reaction pathway allows us to ensure the expected reaction pathway has been followed correctly and also provides key insight into the features of these complexes that stabilize reaction intermediates and result in the key differences between them. It is important to consider that the occupancy/character numbers presented in this section are dependent on the particular method used (functional, etc.). For example, using the TPSSh functional produces IBOs that are visually very similar but are, on the whole, more localized, where a 60:40 character orbital might become a 70:30 instead. We therefore draw information specifically from the way these properties change as the reaction progresses and the differences between the different complexes.

#### 3.3.1 First Reducing Equivalent—Steps [1]–[3]

From the starting structures, certain differences are already evident. The electrons on molybdenum are delocalized very strongly to all of the iron centers, one per iron with the opposite spin. The α electron is delocalized with 57.0% Fe character, the β electrons both with 41.2% Fe character. Overall, the valence d-electrons of the molybdenum effectively form 1-electron sigma bonds to the iron centers. This explains the exceptionally low spin population observed on the Mo (≈−0.3 in the starting structure) despite the presence of unpaired electrons.

There is also a back-bonding interaction accompanying these, with 6.8% and 10.0% Mo characters on the respective back-bonding iron electrons. Even the β electron depicted as shared between the two α iron centers has 5.9% Mo character, with the rest shared equally between the α iron centers. In the isoelectronic vanadium complex, the equivalent α electron is much more localized toward the flipped iron center, with only 30.0% V character, while the β electrons are delocalized only slightly less at 39.9% Fe character each. The back-bonding is less prevalent, with 5.0% V character for each of the three back-bonding iron electrons, and so is the delocalization of the shared β electron, down to 3.3% V character. In the isocharge vanadium complex, the two β electrons on vanadium are delocalized even less, down to 30.2% Fe character, with the back-bonding following suit at 4.0% V character. However, the shared β electron interacts more strongly with the vanadium, at a 12.5% V character.

The equivalent data for relative occupancies after substitution of acetonitrile with hydrazine are presented in Table 2 under M-N_2_H_4_. For the molybdenum complex, we observe little change in the most important interactions upon this substitution (namely, those involving the Mo d-electrons), and the same or slightly larger percentages are observed for the isocharge V complex. However, for the isoelectronic V complex, upon substitution with hydrazine, the delocalization of the α electron drops to 0 (becomes entirely localized on the flipped iron center), and the two β electrons on the vanadium are delocalized with only 27.5% Fe character each, down from 39.9%. This is likely the reason behind the large discrepancy in the energies of the first substitution step in the energy profiles. To further illustrate this, upon formation of a formal positive charge on the ligand in the M-N_2_H_5_ complex, and subsequent reduction in the electron density donated by the ligand to the vanadium, restoration of the V–Fe interaction strength is immediately and clearly observed, with relevant electrons back to near 60:40 ratio of delocalization.

**Table 2:** Calculated Occupancies of Key Fe/M d-Electrons in the Tested Complexes (in %)

orbital	isocharge V	iso-elec V	molybdenum
M-N_2_H_4_			
Fe1-M α	96.0–4.0	96.0–4.0	89.8–10.2
Fe2-M α	96.0–4.0	96.0–4.0	89.8–10.2
Fe3-M α	n/a	100.0–0.0	58.8–41.2
Fe1-M β	35.4–64.6	28.7–71.3	44.4–55.6
Fe2-M β	34.9–65.1	28.2–71.8	43.9–56.1
Fe3-M β	98.5–1.5	91.7–8.3	92.8–7.2
M-N_2_H_5_			
Fe1-M α	92.4–7.6	92.9–7.1	86.7–13.3
Fe2-M α	91.9–8.1	92.4–7.6	86.6–13.4
Fe3-M α	66.8–33.2	61.3–38.7	48.7–51.3
Fe3-M α^a^	n/a	100.0–0.0	98.5–1.5
Fe1-M β	48.7–51.3	42.3–57.7	42.1–57.9
Fe2-M β	48.0–52.0	42.3–57.7	41.6–58.4
Fe3-M β	92.9–7.1	83.9–16.1	78.9–21.1

a Additional electron localized on Fe3 after reduction of the complex.

Upon protonation, in the molybdenum complex, all of the previously mentioned interactions are carried through qualitatively the same, between 0 and 2% more strongly delocalized, in response to slightly smaller electron density incoming from the hydrazine, as would be expected following its protonation. The same is observed for the interactions in the isocharge vanadium complex upon interaction, but of special note are the back-bonding interactions, which become significantly stronger, up to 7.5% for the back-bonding from the β iron, and up to 8.3% for the back-bonding from the α irons. The isoelectronic complex experiences the largest change upon deprotonation, with much stronger interactions in the delocalized vanadium electrons, up to 42.1% Fe character for the β electrons, and up to 36.3% V character for the α electron. The back-bonding is likewise significant, with 6.6% V character from the α irons and 15.2% V character from the β iron.

Upon reduction, in the molybdenum complex, essentially the same occurs as for protonation, with a general slight increase in the extent of delocalization in metal–metal interactions. The new α electron is formally placed on the β iron center, where it is only slightly delocalized to the molybdenum at a 5.7% Mo character. The existing α electron delocalized between the same iron and molybdenum comes even closer to perfect covalency with 54.6% Mo character. Back-bonding from the β iron increases from 7.0 up to 14.8% and stays the same from the α irons. Upon its first reduction, the isocharge complex becomes the initial isoelectronic structure depicted in the table, which can be summarized as the addition of a strongly localized α electron to the β iron and an accompanying decrease in the delocalizations of the two heterometal electrons. In the isoelectronic complex, we once again observe a large change with significantly stronger interaction between the vanadium and iron centers, with vanadium β electrons up to 42.1% Fe character, V–Fe α electron up from 0 to 36.3% V character, and back-bonding from α irons appears at 6.6% Fe character from the α irons while back-bonding from β iron doubles to 15.0%.

After one of each protonation and reduction steps, we arrive at a neutral species with interactions shown in Table 2 under M-N_2_H_5_. At this stage, the complexes are remarkably similar, with the larger extent of back-bonding and previously seen preference for the heterometal over the iron for the Fe3 α electron being the main points of disparity between the isoelectronic Mo/V complexes. The isocharge vanadium complex, while having slightly weaker back-bonding, has almost perfect covalency on the two vanadium β electrons.

#### 3.3.2 Transition State—Step [4] and Beyond

**Figure 8:**
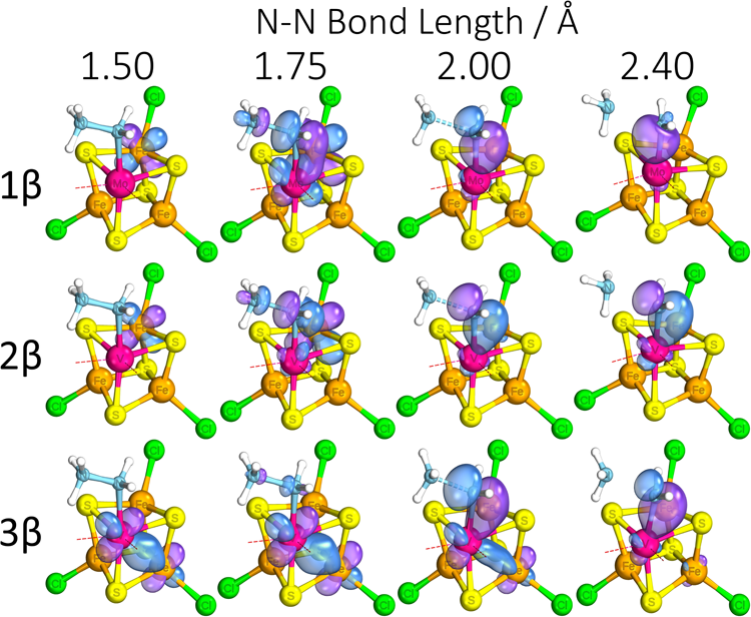
Representative IBOs at given N–N bond lengths (in Å) showing transfer of a reducing β electron upon N–N bond cleavage. Labels 1, 2, and 3 correspond to [MoFe_3_S_4_]^3+^, [VFe_3_S_4_]^2+^, and [VFe_3_S_4_]^3+^, respectively. Atoms: magenta = Mo/V, cyan = N, yellow = S, orange = Fe, red = O, green = Cl, and white = H. Part of the structure has been omitted for clarity.

**Figure 9:**
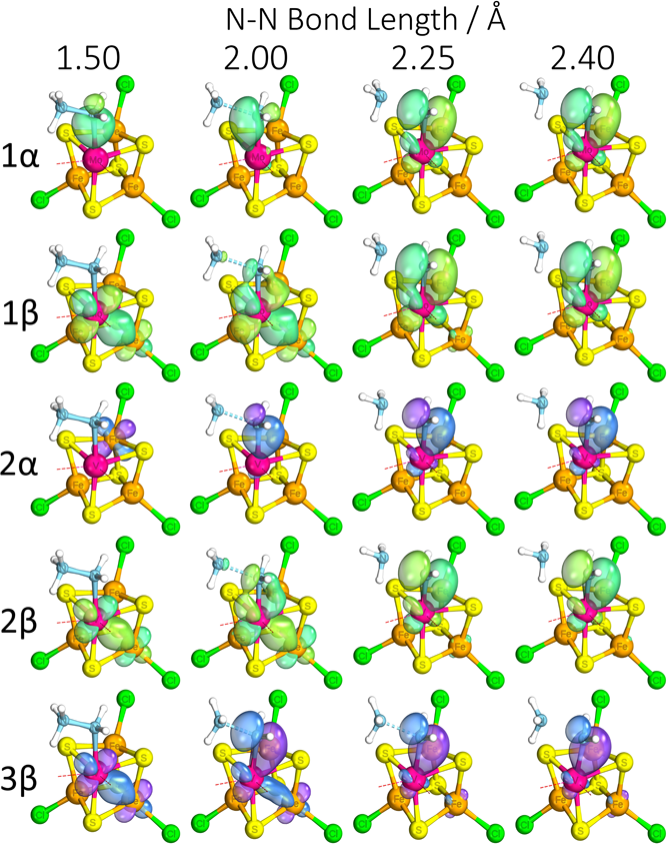
Representative IBOs at given N–N bond lengths (in Å) showing the formation of stabilizing M–N π-bond in the molybdenum complex and its equivalents in the vanadium complexes. α and β electrons labeled accordingly. Labels 1, 2, and 3 correspond to [MoFe_3_S_4_]^3+^, [VFe_3_S_4_]^2+^, and [VFe_3_S_4_]^3+^, respectively. Atoms: magenta = Mo/V, cyan = N, yellow = S, orange = Fe, red = O, green = Cl, and white = H. Part of the structure has been omitted for clarity.

**Figure 10:**
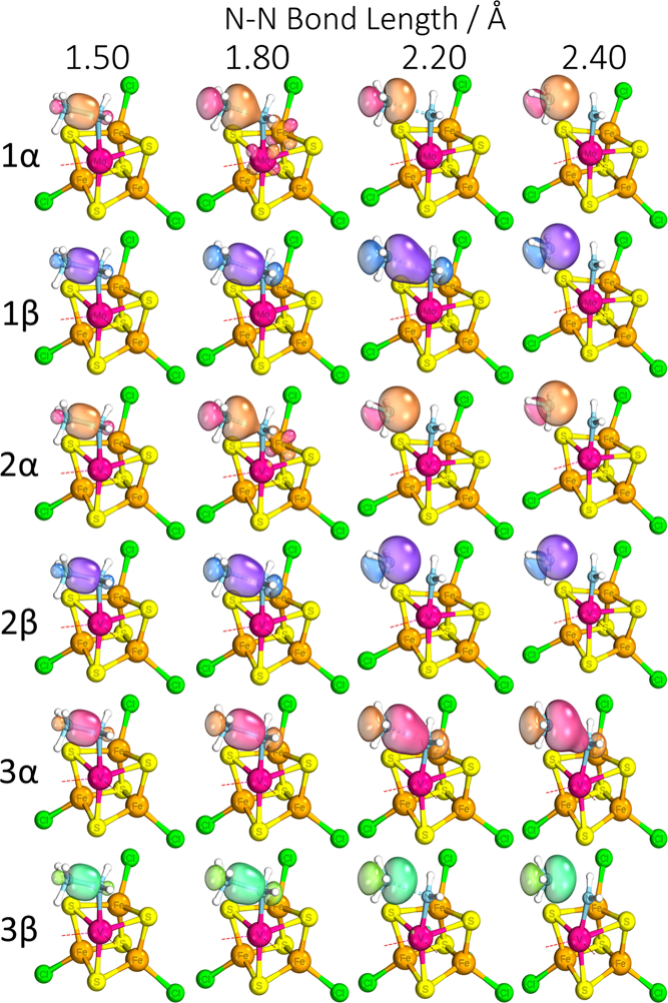
Representative IBOs at given N–N bond lengths (in Å) showing breakage of the N–N σ bond over the transition state. α and β electrons labeled accordingly. Labels 1, 2, and 3 correspond to [MoFe_3_S_4_]^3+^, [VFe_3_S_4_]^2+^, and [VFe_3_S_4_]^3+^, respectively. Atoms: magenta = Mo/V, cyan = N, yellow = S, orange = Fe, red = O, green = Cl, and white = H. Part of the structure has been omitted for clarity.

The transition-state search started with this intermediate, and the scans and accompanying key IBO changes are presented in Figures 8–10. Despite the similarities in energy profiles of the isocharge vanadium and molybdenum catalyzed reactions, the transition state occurs at notably different bond distances: 1.99 Å for isocharged vanadium, 1.74 Å for isoelectronic vanadium, and 1.78 Å for molybdenum, corresponding to bond stretches from minima by factors of 1.38, 1.20, and 1.23 respectively. This is best observed visually in Figure 8, which shows transfer of the reducing electron over the transition state. Other than showing the importance of the Mo/V center in facilitating a smooth, gradual transfer of reducing equivalents to the product ligand as required, we can qualitatively observe the larger involvement of the Mo center as an intermediary for electron density during this transition, as compared to its isoelectronic vanadium counterpart. While IBOs best describe this electron transfer as depicted, due to the Mo–Fe delocalized electron remaining constant at the start and the end of the transition state, the overall movement is better thought of as the shift of the Mo–Fe electron into the hydrazine as the N–N bond breaks, followed by the electron facilitating the Mo–Fe interaction by the electron which was localized on the Fe3 center upon reduction of the complex, as is depicted in the bottom row of Figure 8, due to the lack of that localized electron and therefore lack of one of the V–Fe interactions in the post-TS complex.

The molybdenum appears to stabilize the Mo–NH_2_ intermediate shortly after N–N bond cleavage via the formation of a strong π-bonding interaction, formed with an electron formerly delocalized to one of the iron centers (Figure 9). Most notably, the vanadium complexes did not show this interaction. While we can observe movement of the same two electrons down the same paths in the isoelectronic vanadium complex, the resulting interaction does not reach the proper π-bond configuration, and the isocharged vanadium complex only dedicates one electron to the equivalent interaction by the end, and even that still retains non-negligible delocalization back to the Fe the electron came from.

Further insight can be gained by inspecting the orbitals associated with N–N bond cleavage (Figure 10). Between the isoelectronic complexes, despite their similar transition-state bond lengths, the complete dissociation of the NH_3_ lone pair occurs earlier for the vanadium complex than the molybdenum, with the latter maintaining a level of delocalization on the β electron for a significant length of the N–N bond scan past the transition state and N–N bond cleavage. Between the isocharged complexes, the same effect is observed on both, although the vanadium complex prefers to maintain an α electron delocalized instead, which makes sense given its two-β electron configuration with no α electron between V–Fe3. As the electron configuration of the isocharged complex requires N–N cleavage to result in the formal oxidation of Fe3 to +3, the transition-state bond length ends up longer.

Looking at the overall picture presented by IBOs over the intermediates, as well as the N–N relaxed surface scan, molybdenum displays greater flexibility in the electronic structures it is capable of assuming for stability by better utilizing electrons in its M–Fe bonds, which vanadium, particularly at larger electron densities, is not capable of achieving. Outside of the transition state, electrons within the M–Fe–S system are generally more delocalized in the molybdenum complex compared to the vanadium ones and are able to fluctuate more strongly between intermediates, allowing for this flexibility. On the other hand, the vanadium’s system of delocalized electrons is less extensive and evidently capable of supporting less electron density in this network, prone to significantly withdraw parts of it upon addition of more reducing equivalents, as depicted in the example of the acetonitrile for hydrazine substitution.

The observations with regard to relative trends of electron movements are effectively mirrored for the second reducing equivalent.

### 3.4 Alternative Binding Sites for N_2_H_4_

While it has been quite convincingly proven that the studied complex properly binds hydrazine onto Mo rather than anywhere else, there is no such decisive evidence yet for the binding site of N_2_ and other reaction intermediates in the nitrogenase cofactors. In fact, it is presently thought to be more likely that the binding occurs on the iron centers, without direct involvement of the Mo/V.^7,38,39^ It therefore seems prudent to consider how the differing complexes studied here handle binding hydrazine at the Fe centers as well, potentially further shedding some light on the difference in different nitrogenases and their substrate bindings. To that end, we optimized a series of structures with the acetonitrile still in place on the Mo/V center and hydrazine bound instead to various Fe centers, both as a terminal and bridging ligand.

#### 3.4.1 Binding Energies

In all cases, the binding of hydrazine to the iron centers is unfavorable, with the smallest energy barrier among all complexes being 5.2 kcal mol^–1^. The terminal binding of the hydrazine is preferable in all cases. For either of the vanadium complexes, it was not possible to optimize structures with the hydrazine as a bridging ligand, as it either naturally optimized itself back to a terminal ligand or converged to a wildly incorrect electronic state to maintain bridging (even in the latter case, at a cost of +14.1–35.4 kcal mol^–1^ over the terminal geometry). In the molybdenum complexes, it is possible to maintain the bridging configuration at the correct electronic state, but only as a bridge between the two α iron centers, and this is at least 8.7 kcal mol^–1^ uphill from the terminal geometry. In the case of bridging between an α and β iron center, the geometry spontaneously optimizes to have the hydrazine to be a terminal ligand to the α iron instead.

The results in terms of energetics are summarized in Table 3. In the molybdenum complex, the hydrazine prefers to bind to the α irons, in the isoelectronic vanadium complex to the β iron, and without a meaningful preference in the isocharge vanadium complex. Although still unfavorable, hydrazine would find it easier to bind to the irons in the molybdenum complex by at least 1.8 kcal mol^–1^.

**Table 3:** Relative Absolute Free Energies and Binding Free Energies for the Complexes with Hydrazine Bound to the Indicated Metal Centers, with Acetonitrile Solvent Ligated to the Mo/V Center^a^

center	relative *E*	binding Δ*G*
[MoFe_3_S_4_]^3+^		
Mo	n/a	–9.91
Fe1	0.00	5.23
Fe2	0.36	5.58
Fe3	2.53	7.76
Fe1–Fe2	11.19	16.42
[VFe_3_S_4_]^3+^		
V	n/a	–8.69
Fe1	0.00	7.40
Fe2	0.06	7.46
Fe3	0.81	8.21
[VFe_3_S_4_]^2+^		
V	n/a	11.39
Fe1	1.70	8.71
Fe2	1.09	8.10
Fe3	0.00	7.01

a Binding Δ*G* for the Mo/V centers represents the energy for substitution of acetonitrile. All energies are given in kcal mol^–1^.

#### 3.4.2 IBO Analysis—[MoFe_3_S_4_]^3+^

Analyzing the IBOs of the structures in the molybdenum complex, the binding of hydrazine occurs largely via the interaction of one electron only, the one opposite to the spin of the iron in question (relevant IBOs for this can be found in the Supporting Information). There is a 12–13% Fe character on the opposing electron and 3–4% Fe character on the electron matching that of the iron, in contrast to the 12–13% Mo character observed for both lone pair electrons in the hydrazine–Mo bond. This makes sense, as the molybdenum has interactions with both types of electrons to three other metal centers and can effectively stabilize both α and β spins appropriately, whereas the iron centers only significantly interact with the molybdenum, and at most one other iron center in the case of the two α irons. Upon binding of hydrazine to one of the α iron centers, the extent of delocalization of the β electrons from the Mo to the relevant iron center decreases (from 43.5 to 35.7% Fe character in the case of Fe1 binding). Additionally, the β electron formerly equally delocalized between the two α iron centers becomes more strongly pulled toward the iron with the bound hydrazine, which appears counterintuitive at first, but the relatively small deformation of the bond here allows the β electron in question to capitalize more strongly on the larger deformation of the Fe–Mo bond.

In the case of binding on the β iron, there are only minor changes to the electronic structure of the complex, most notable of which is once again the extent of delocalization of the β electrons to the α irons, likely due to the now larger presence of α electrons on the β iron as opposed to just on the α ones. It seems reasonable to deduce that the preference for binding on the α irons, in this case, stems from the extra versatility of charge stabilization of the α irons stemming from their additional shared electron with another iron center. In the case of the ligand bridging two α irons, each nitrogen lone pair binds to an iron with 8.9% Fe character on the β electron and 2.9% Fe character on the α electron. While there are once again no major changes in the electronic structure, noticeable stabilizing effects include a decrease in α back-bonding from the α irons to the Mo from 10.1 to 8.3%, a slight decrease to the already small interaction between the β iron and the α irons, and a stronger localization of the shared β electron almost entirely just among the two α iron centers versus the previous 6.3% Mo character. Overall, the antiferromagnetic interaction shifts slightly away from the molybdenum and toward the new ligand, albeit not significantly, which is to be expected with the relatively weak nature of the bond. The decrease in overall intrametal interaction required to stabilize the bridging ligand here is evidently costly and results in this mode being the least favorable.

#### 3.4.3 IBO Analysis—[VFe_3_S_4_]^3+^

Moving onto the isocharged vanadium complex, curiously, binding of the hydrazine ligand onto α iron centers occurs with both electrons even with the terminal configuration, with 17.9% Fe character and 14.9% Fe character on the α and β lone pair electrons, respectively. Most notably, the favored electron configuration now has the β iron formally reduced by the iron binding the hydrazine, with the electron being strongly localized on the now +2 β iron. Aside from this, a major stabilizing factor for the additional α electron density is a large distortion of the β electron shared between the α irons, toward the Fe with the hydrazine ligand, from 42.5:42.3:14.55% Fe/Fe/V character to 73.4:20.5:4.4%. This is a much larger change in the localization of this electron compared to that observed in any Mo complex counterparts. The stabilizing factor for the addition of the β electron density is decrease of β electron delocalization from the V to the Fe in question, accompanied by a decrease in the α back-bonding from the same Fe from 4.2 to 1.05% V character and an increase in β back-bonding from the β Fe to the V from negligible to 6.15% V character. Binding on the β Fe, however, occurs with majority α character once more with 11.9 and 4.0% α and β lone pair electrons, respectively. The same reduction of the β iron is observed once again, but this time with the new electron delocalized between the iron and vanadium centers with a 31% V character. The two α irons’ shared electron loses all V character, and the vanadium’s two β electrons interact more strongly with the α irons, from 63.33% V character to 43.15% V character. Overall, the isocharge complexes have to undergo much larger changes in their electronic structure to accommodate binding of these ligands in comparison to their Mo counterparts but benefit from the flexibility of an easily reducible iron center to make the changes needed for stability.

#### 3.4.4 IBO Analysis—[VFe_3_S_4_]^2+^

Lastly, we examined the isoelectronic vanadium complex. Binding of hydrazine onto the irons occurs in essentially the same way with the same % characters as in the molybdenum complex. Unlike the molybdenum complex, the vanadium delocalizes both of its β electrons more, from 69.8% V character to 59.4%, and the previously localized α electron on the β iron becomes localized to the vanadium with 31.1% V character. The shared β electron gains 4% V character. For the β Fe binding, we observe a decrease in delocalization to and from the β iron, compensated by a familiar increase in delocalization between vanadium and the two α irons, all to a similar extent as with the α iron binding.

Overall, the complexes certainly share and employ many of the same features for stability, with the major difference in this case being the β iron. In Mo complexes, this center starts off with a covalent 1-electron bond with the molybdenum; in V complexes, this center has no major interactions with the other metals, its α electron either absent or strongly localized. With this in mind, the relative preferences for the α/β binding of hydrazine make sense. With Mo, any binding to the β Fe disrupts the α Mo–Fe interaction, whereas, with V, this interaction is not present at the start and can yet be utilized for stability. Overall, it is remarkable that the vanadium complexes, having such distinctly different energy profiles for the reaction discussed earlier, are able to nonetheless exploit the versatility of this electronic structure to arrive at essentially the same binding energy within error. Regardless, Mo’s lower binding energy and comparative ability to adapt to a bridging ligand provide further evidence toward the reason behind Mo nitrogenase’s superior efficacy to its vanadium counterpart.

We have also looked into the possibility of hydrazine binding as a bridging ligand between one iron center and the Mo/V centers, with and without acetonitrile solvent ligated. In all optimized geometries with the acetonitrile still ligated, the hydrazine optimized to a terminal M-N_2_H_4_ conformation instead, substituting either one of the oxygens (if attempted bridge between Fe1/Fe2-M) or the acetonitrile (if attempted bridge between Fe3-M). Similar behavior is observed for the molybdenum and isoelectronic vanadium complexes without acetonitrile ligated. The isocharged complex does optimize toward a bridging Fe1/Fe2-M hydrazine, but this corresponds to a >34 kcal mol^–1^ binding Δ*G*, making it a nonviable option relative to the previously investigated binding modes.

## 4 Conclusions

An in-depth investigation of the electronic structure of nitrogenase-adjacent compounds demonstrates clear differences in the way the electrons are stored and how they move as the reduction reaction progresses. On the whole, the vanadium in its complexes displays a distinctly poorer ability to stabilize intermediates via electron delocalization across the M–Fe–S framework and to store and transfer reducing equivalents. This is quite clearly evident from both the IBO analyses and the energy profiles for the isoelectronic Mo/V complexes. Given the Mo/V center’s likely involvement in intermediate stabilization via similar intracomplex interactions with the Fe centers in the nitrogenase enzymes, it is not unreasonable to suppose that the lower efficacy of the vanadium-dependent nitrogenase may be in part due to this effect, which is further supported by observations made in prior work on the ground-state molybdenum and vanadium-dependent cofactors.^40^ Although the isocharge vanadium complex may appear close or marginally superior to the molybdenum if you only consider the energy profiles, we must consider it is only 1 electron away from the isoelectronic energy profile, and while one electron is all that is needed per molecule of NH_3_ in the case of hydrazine reduction, that is not going to be the case for N_2_, where storage and utilization of many reducing equivalents simultaneously may well be required. In addition, the alternate electronic state in the isocharge complex assumes the missing electron came from the part of a nitrogenase cofactor, which is modeled by the cubane, rather than the remainder of the Fe–S framework. Previous work done with quantum mechanics/molecular mechanics (QM/MM) has suggested that the electron in question may come precisely from the part of the cofactor not included in this particular model, leaving the model as depicted in the isoelectronic configuration.^41^ Should binding indeed occur on the iron centers rather than the heterometal in the nitrogenase cofactors, the evidence for its lower efficacy still remains even with this model, and the reasoning why should be largely the same, with molybdenum being more flexible and able to undertake required changes for new ligands to the same effect with less profound adjustments to its electronic structure.

## Supporting Information Available

Additional data, including a high spin/broken-symmetry geometry comparison; spin/charge populations; energies of all structures; additional IBOs; and Cartesian coordinates of all optimized geometries (PDF)
