# Supplementary material for: Computational Study on the Influence of Mo/V Centers on the Electronic Structure and Hydrazine Reduction Capability of [MFe3S4]3+/2+ Complexes
Source: Inorg Chem. 2023 Sep 27;62(40):16401–11. doi: 10.1021/acs.inorgchem.3c02072 (PMC10565805; doi:10.1021/acs.inorgchem.3c02072)
Supplement: Supplementary file 1 — ic3c02072_si_001.pdf [file ic3c02072_si_001.pdf]

Supporting Information

Computational study on the influence of Mo/V centres on the electronic structure and hydrazine reduction capability of $[MFe_3S_4]^{3+/2+}$ complexes.

Maxim Barchenko,[§#] Thomas Malcomson,[#] Sam P. de Visser,[§⊥] and Patrick J. O'Malley.[*#]

[§] Manchester Institute of Biotechnology, The University of Manchester, 131 Princess Street, Manchester M1 7DN, United Kingdom

[#] Department of Chemistry, The University of Manchester, Oxford Road, Manchester M13 9PL, United Kingdom

[⊥] Department of Chemical Engineering, The University of Manchester, Oxford Road, Manchester M13 9PL, United Kingdom

Email: patrick.o'malley@manchester.ac.uk

Methods

Unless explicitly stated otherwise in a relevant caption, all data presented in this document has been calculated with the ORCA software package version 4.2.1. DFT calculations were done with the BP86 functional, def2-tzvp basis set on all atoms, D3BJ dispersion correction, and implicit solvation in acetonitrile described with the CPCM-SMD model.

Table S1 – Comparison of optimised geometries for the high spin and broken symmetry states of the $MoFe_3S_4$ complex.

**Spin State**	**Mo-Fe1 Distance**	**Mo-Fe2 Distance**	**Mo-Fe3 Distance**	**Fe1-Fe2 Distance**	**Fe1-Fe3 Distance**	**Fe2-Fe3 Distance**
**M_S = 8.5**	3.25	3.24	3.26	2.78	2.88	2.88
**M_S = 1.5**	2.75	2.75	2.84	2.93	2.88	2.88

Table S2 – Absolute, Zero Point, and Gibbs Free Energies (in au) of optimised structures for the investigated hydrazine reduction pathway.

Structure	E [au]	ZPE [au]	G [au]
[Mo]-ACN	-9188.190451	0.110375	-9188.142914
[Mo]-N_2H_4	-9167.321451	0.120096	-9167.262440
[Mo]-$N_2H_5^+$	-9167.777021	0.133089	-9167.704920
[Mo]-$N_2H_4^-$	-9167.430593	0.118782	-9167.373383
[Mo]-N_2H_5	-9167.889242	0.128731	-9167.824086
[Mo]-N_2H_5(TS)	-9167.873586	0.124424	-9167.811433
[Mo]-NH_2	-9111.317899	0.086457	-9111.292307
[Mo]-NH_3^+	-9111.804749	0.099658	-9111.766168
[Mo]-NH_2^-	-9111.450757	0.084953	-9111.427564
[Mo]-NH_3	-9111.969060	0.099983	-9111.930451
$[V]^{2+}$-ACN	-10064.134926	0.109151	-10064.087995
$[V]^{2+}$-N_2H_4	-10043.226573	0.116592	-10043.173573
$[V]^{2+}$-$N_2H_5^+$	-10043.728272	0.128104	-10043.661529
$[V]^{2+}$-$N_2H_4^-$	-10043.337103	0.113794	-10043.286694
$[V]^{2+}$-N_2H_5	-10043.817793	0.126592	-10043.754351
$[V]^{2+}$-N_2H_5(TS)	-10043.804523	0.123301	-10043.744647
$[V]^{2+}$-NH_2	-9987.267097	0.084922	-9987.243166
$[V]^{2+}$-NH_3^+	-9987.777966	0.098960	-9987.741291
$[V]^{2+}$-NH_2^-	-9987.370035	0.083788	-9987.348290
$[V]^{2+}$-NH_3	-9987.909358	0.097966	-9987.873428
$[V]^{3+}$-ACN	-10063.997090	0.108617	-10063.953607
$[V]^{3+}$-N_2H_4	-10043.127314	0.118492	-10043.071189
$[V]^{3+}$-$N_2H_5^+$	-10043.588537	0.130529	-10043.519547
$[V]^{3+}$-$N_2H_4^-$	-10043.258150	0.115319	-10043.206583
$[V]^{3+}$-N_2H_5	-10043.728307	0.128187	-10043.662674
$[V]^{3+}$-N_2H_5(TS)	-10043.701969	0.123356	-10043.641731
$[V]^{3+}$-NH_2	-9987.133177	0.086456	-9987.107372
$[V]^{3+}$-NH_3^+	-9987.622119	0.099764	-9987.582790

$[V]^{3+}$-NH_2^-	-9987.268081	0.085094	-9987.243401
$[V]^{3+}$-NH_3	-9987.777861	0.099005	-9987.740815

Table S3 – Mulliken Spin and Charge populations on Fe/Mo/V centres of the optimised geometries for the structures involved in the hydrazine reduction mechanism and featured on the energy profile figures.

	Spin				Charge			
Structure	Fe1	Fe2	Fe3	Mo/V	Fe1	Fe2	Fe3	Mo/V
[Mo]-ACN	3.05	3.06	-3.09	-0.30	0.42	0.42	0.42	0.52
[Mo]-N_2H_4	3.06	3.06	-3.09	-0.33	0.42	0.42	0.41	0.57
[Mo]-$N_2H_5^+$	3.05	3.04	-3.08	-0.29	0.42	0.42	0.41	0.55
[Mo]-$N_2H_4^-$	3.11	3.11	-2.54	-0.30	0.45	0.44	0.39	0.52
[Mo]-N_2H_5	2.95	2.94	-2.14	-0.33	0.45	0.45	0.40	0.45
[Mo]-N_2H_5(TS)	2.96	2.98	-2.53	-0.22	0.45	0.45	0.40	0.50
[Mo]-NH_2	3.27	2.84	-2.76	-0.06	0.47	0.41	0.37	0.74
[Mo]-NH_3^+	3.00	3.00	-2.74	-0.21	0.42	0.42	0.37	0.56
[Mo]-NH_2^-	2.93	2.89	-2.86	-0.30	0.45	0.44	0.43	0.60
[Mo]-NH_3	2.87	2.87	-2.81	-0.26	0.43	0.43	0.40	0.56
$[V]^{2+}$-ACN	3.17	3.17	-3.11	-0.69	0.40	0.40	0.40	0.42
$[V]^{2+}$-N_2H_4	3.25	3.25	-2.85	-1.46	0.43	0.41	0.37	0.57
$[V]^{2+}$-$N_2H_5^+$	2.99	2.98	-2.89	-0.51	0.40	0.36	0.35	0.57
$[V]^{2+}$-$N_2H_4^-$	3.08	3.09	-2.18	-0.78	0.42	0.39	0.36	0.51
$[V]^{2+}$-N_2H_5	3.09	3.07	-2.19	-0.72	0.42	0.37	0.33	0.53
$[V]^{2+}$-N_2H_5(TS)	3.06	3.07	-2.47	-0.58	0.42	0.38	0.34	0.55
$[V]^{2+}$-NH_2	3.02	3.28	-2.89	-0.17	0.43	0.44	0.39	0.45
$[V]^{2+}$-NH_3^+	2.96	3.19	-2.84	-0.10	0.39	0.41	0.37	0.48
$[V]^{2+}$-NH_2^-	3.03	3.04	-2.92	-0.69	0.41	0.41	0.37	0.44
$[V]^{2+}$-NH_3	3.01	3.01	-2.90	-0.59	0.40	0.40	0.39	0.50
$[V]^{3+}$-ACN	3.16	3.16	-3.28	-1.31	0.41	0.41	0.44	0.39
$[V]^{3+}$-N_2H_4	3.16	3.16	-3.30	-1.29	0.41	0.38	0.40	0.58
$[V]^{3+}$-$N_2H_5^+$	2.36	2.39	-2.73	-0.17	0.34	0.30	0.33	0.57
$[V]^{3+}$-$N_2H_4^-$	3.00	3.00	-2.90	-0.57	0.40	0.38	0.36	0.55
$[V]^{3+}$-N_2H_5	2.99	2.98	-2.89	-0.51	0.40	0.36	0.35	0.57
$[V]^{3+}$-N_2H_5(TS)	2.98	2.99	-2.88	-0.25	0.39	0.38	0.36	0.56
$[V]^{3+}$-NH_2	2.80	2.46	-2.75	0.07	0.40	0.37	0.37	0.46
$[V]^{3+}$-NH_3^+	2.41	2.52	-2.68	-0.23	0.34	0.35	0.36	0.46
$[V]^{3+}$-NH_2^-	3.28	3.02	-2.89	-0.17	0.44	0.42	0.38	0.45
$[V]^{3+}$-NH_3	2.97	3.18	-2.84	-0.13	0.39	0.41	0.37	0.48

Table S4 – Absolute, Zero Point, and Gibbs Free Energies (in au) of optimised structures for the alternative hydrazine binding sites.

Structure	E [au]	ZPE [au]	G [au]
[Mo]- Fe1Fe2 Bridging	-9300.115871	0.160743	-9300.022417
[Mo]-Fe1 Terminal	-9300.135581	0.162710	-9300.040248
[Mo]-Fe2 Terminal	-9300.135573	0.162861	-9300.039679
[Mo]-Fe3 Terminal	-9300.132177	0.162147	-9300.036219
$[V]^{2+}$-Fe1 Terminal	-10176.089270	0.162040	-10175.993204
$[V]^{2+}$-Fe2 Terminal	-10176.089356	0.162141	-10175.994180
$[V]^{2+}$-Fe3 Terminal	-10176.089644	0.163027	-10175.995920
$[V]^{3+}$-Fe1 Terminal	-10175.897868	0.163002	-10175.802706
$[V]^{3+}$-Fe2 Terminal	-10175.944458	0.163026	-10175.847027
$[V]^{3+}$-Fe3 Terminal	-10175.940563	0.162727	-10175.845827

Table S5 – Mulliken Spin and Charge populations on Fe/Mo/V centres of the optimized geometries for the alternative hydrazine binding sites.

	Spin				Charge			
Structure	Fe1	Fe2	Fe3	Mo/V	Fe1	Fe2	Fe3	Mo/V
[Mo]-Bridging Fe1Fe2	3.13	3.12	-3.11	-0.33	0.39	0.41	0.43	0.51
[Mo]-Fe1 Terminal	3.08	3.05	-3.05	-0.34	0.36	0.39	0.39	0.54
[Mo]-Fe2 Terminal	3.05	3.08	-3.05	-0.35	0.39	0.36	0.39	0.53
[Mo]-Fe3 Terminal	3.04	3.05	-3.15	-0.30	0.39	0.41	0.34	0.53
$[V]^{2+}$-Fe1 Terminal	3.20	3.16	-3.09	-0.76	0.38	0.36	0.37	0.45
$[V]^{2+}$-Fe2 Terminal	3.17	3.20	-3.09	-0.76	0.36	0.38	0.37	0.44
$[V]^{2+}$-Fe3 Terminal	3.17	3.19	-3.25	-0.68	0.36	0.38	0.40	0.42
$[V]^{3+}$-Fe1 Terminal	2.10	3.18	-3.07	-0.44	0.19	0.38	0.36	0.48
$[V]^{3+}$-Fe2 Terminal	3.18	2.10	-3.07	0.45	0.38	0.19	0.36	0.47
$[V]^{3+}$-Fe3 Terminal	2.73	2.57	-3.09	-0.42	0.33	0.36	0.33	0.42

Table S6 – Absolute, Zero Point, and Gibbs Free Energies (in au) of optimised supplementary structures.

Structure	E [au]	ZPE [au]	G [au]
Cobaltocene	-1770.307898	0.160905	-1770.182230
Cobaltocene+	-1770.187591	0.165625	-1770.055586
Lutidinium Acid	-327.530510	0.152588	-327.410529
Lutidine	-327.072136	0.138768	-326.965448
Hydrazine	-111.936370	0.051557	-111.907497
Ammonia	-56.594097	0.033131	-56.580061
Acetonitrile	-132.8211054	0.043772	-132.801355

Table S7 – J couplings for tested broken symmetry states of $[MoFe_3S_4]^{3+}$. The values presented here were calculated with the TPSSh functional and ZORA relativistic approximation with the ZORA-def2-tzvp basis set, but similar results were obtained with our usual method, and without ZORA. Centres 1, 2, and 3 are irons, 4 is molybdenum. We note this data has limited meaningfulness given the extensive delocalisation of electrons/mixed valence in the system but are presented here for completion regardless.

State	Spin	E(HS)-E(BS) / cm^{-1}
BS1	2.5	19381.9
BS2	3.5	8817.1
BS3	2.5	18815.0
BS4	6.5	12583.6
BS5	1.5	22623.5
BS6	2.5	8852.2
BS7	1.5	23313.7
J Coupling	Coupling /cm^{-1}	
J12	-62	
J13	-539	
J14	-694	
J23	-65	
J24	-1268	
J34	-568	
Coupling-Derived Ground Spin State	1.5	
Closest-Lying Excited Spin State	2.5	

Table S8 – Single point TPSSh absolute energies (in au) of the BP86-optimised structures for the investigated hydrazine reduction pathway.

Structure	E [au]
[Mo]-ACN	-9187.124174
[Mo]-N_2H_4	-9166.242578
[Mo]-$N_2H_5^+$	-9166.700626
[Mo]-$N_2H_4^-$	-9166.352032
[Mo]-N_2H_5	-9166.815048
[Mo]-N_2H_5(TS)	-9166.790441
[Mo]-NH_2	-9110.244618
[Mo]-NH_3^+	-9110.732518
[Mo]-NH_2^-	-9110.375389
[Mo]-NH_3	-9110.897070
Acetonitrile	-132.825354
Cobaltocene	-1770.130889

Cobaltocene(+)	-1770.016189
Hydrazine	-111.926097
Lutidinium Acid (Protonated)	-327.561329
Lutidine	-327.094355
Ammonia	-56.588027

Table S9 – Single point TPSSh Mulliken Spin and Charge populations on Fe/Mo/V centres of the BP86-optimised geometries for the investigated hydrazine reduction pathway.

	Spin				Charge			
Structure	Fe1	Fe2	Fe3	Mo	Fe1	Fe2	Fe3	Mo
[Mo]-ACN	3.39	3.39	-3.46	-0.59	0.56	0.56	0.57	0.37
[Mo]-N_2H_4	3.39	3.38	-3.45	-0.60	0.56	0.56	0.55	0.39
[Mo]-$N_2H_5^+$	3.39	3.37	-3.46	-0.55	0.56	0.56	0.56	0.37
[Mo]-$N_2H_4^-$	3.38	3.39	-3.04	-0.40	0.58	0.58	0.55	0.33
[Mo]-N_2H_5	3.27	3.26	-2.78	-0.41	0.58	0.58	0.55	0.26
[Mo]-N_2H_5(TS)	3.29	3.29	-2.91	-0.37	0.58	0.58	0.54	0.27
[Mo]-NH_2	3.55	3.26	-3.28	-0.15	0.60	0.54	0.51	0.60
[Mo]-NH_3^+	3.43	3.43	-3.22	-0.34	0.56	0.56	0.51	0.39
[Mo]-NH_2^-	3.29	3.25	-3.31	-0.52	0.58	0.56	0.57	0.44
[Mo]-NH_3	3.24	3.25	-3.29	-0.48	0.56	0.56	0.54	0.38

Figure S1 – IBOs showing 1-electron binding of hydrazine to Fe2 and Fe3 sites.

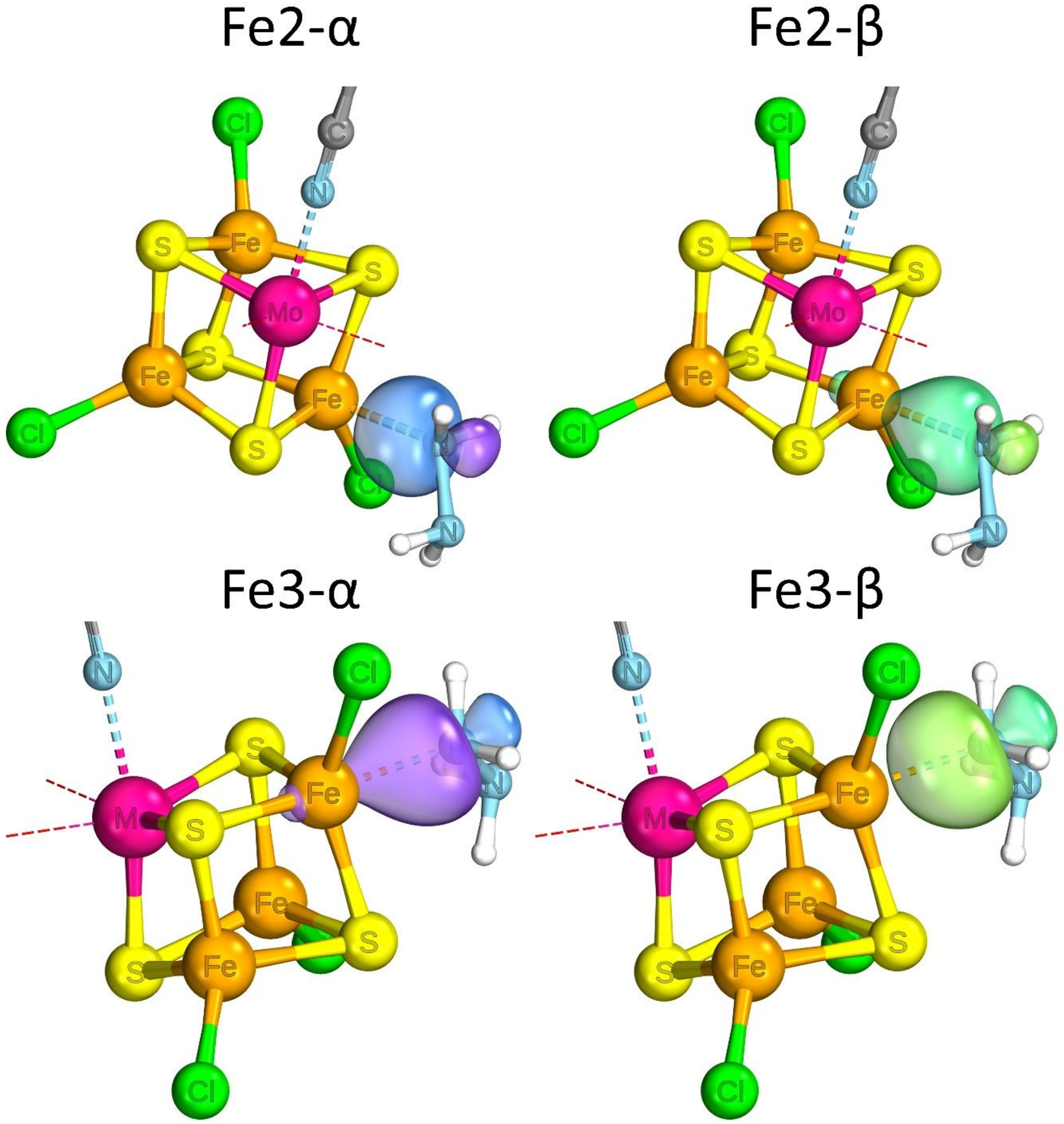

Cartesian coordinates of optimized structures

[Mo]-Acetonitrile

Mo	3.301455000	5.030998000	7.759124000
Fe	2.939866000	2.304661000	7.665724000
Fe	4.991423000	3.553346000	9.349909000
Fe	5.300441000	3.464517000	6.484682000
S	3.087166000	3.640264000	5.861715000
S	2.702890000	3.624508000	9.529977000
S	5.648775000	5.198805000	7.964006000
S	5.220058000	1.719336000	7.876351000
Cl	6.066618000	3.418103000	11.287907000
Cl	1.487749000	0.631465000	7.522628000
Cl	6.676398000	3.522775000	4.755348000
C	0.926023000	6.648489000	8.197810000
C	1.922463000	7.257909000	9.020602000
C	-0.370865000	7.183192000	8.173823000
O	3.123252000	6.711132000	8.989304000
Cl	-1.554161000	6.416251000	7.154168000
C	1.586984000	8.381064000	9.791864000
C	-0.698239000	8.311914000	8.953241000
Cl	2.828992000	9.098941000	10.776963000
Cl	-2.311897000	8.955211000	8.908610000
C	0.280127000	8.910410000	9.761809000
Cl	-0.112186000	10.301313000	10.726908000
O	1.308807000	5.601362000	7.491768000
C	3.738703000	8.614767000	4.554791000
H	4.043190000	9.529891000	5.081367000
H	2.763115000	8.779652000	4.076731000
H	4.483813000	8.376415000	3.783079000
C	3.641219000	7.523683000	5.492536000
N	3.565266000	6.644986000	6.242454000

[Mo]-N_2H_4

Mo	3.544494000	5.664616000	7.419429000
Fe	2.977341000	3.032994000	6.926743000
Fe	4.832220000	3.875460000	9.037705000
Fe	5.582216000	4.086352000	6.255487000
S	3.514655000	4.550705000	5.343047000
S	2.553142000	4.136543000	8.896551000
S	5.830582000	5.603726000	7.978343000
S	5.150456000	2.210982000	7.381650000
Cl	5.597926000	3.413930000	11.069476000
Cl	1.440538000	1.537713000	6.350373000
Cl	7.202638000	4.253350000	4.760702000
C	1.225869000	7.396348000	7.780922000
C	2.127188000	7.793196000	8.812579000
C	-0.036184000	7.998737000	7.691884000
O	3.312249000	7.207574000	8.817946000
Cl	-1.088914000	7.505286000	6.398168000
C	1.729998000	8.777642000	9.730669000
C	-0.428070000	8.984420000	8.620440000
Cl	2.859028000	9.245478000	10.968064000
Cl	-2.001515000	9.709871000	8.499234000
C	0.455092000	9.372237000	9.639971000
Cl	-0.018821000	10.580868000	10.794187000
O	1.669965000	6.478978000	6.931322000
N	3.304590000	7.949227000	5.075069000
H	3.580564000	7.395290000	4.257116000
H	2.378168000	7.576368000	5.332354000
N	4.173490000	7.493314000	6.160118000
H	4.253932000	8.273179000	6.822802000
H	5.111065000	7.369694000	5.766771000

[Mo]-$N_2H_5^+$

Mo	3.596564000	5.684256000	7.411337000
Fe	2.960789000	3.068681000	6.920352000
Fe	4.800781000	3.894485000	9.063288000
Fe	5.603503000	4.045649000	6.286490000
S	3.566990000	4.557231000	5.331719000
S	2.533959000	4.222667000	8.858726000
S	5.864988000	5.579134000	7.996173000
S	5.095151000	2.204176000	7.430450000
Cl	5.480208000	3.431776000	11.111089000
Cl	1.386072000	1.625191000	6.362993000
Cl	7.254337000	4.142479000	4.833363000
C	1.270460000	7.449286000	7.732925000
C	2.157556000	7.804073000	8.782740000
C	-0.003531000	8.020230000	7.662328000
O	3.360427000	7.249403000	8.762101000
Cl	-1.030471000	7.577699000	6.333603000
C	1.735512000	8.726483000	9.754113000
C	-0.424096000	8.940238000	8.642986000
Cl	2.847080000	9.149941000	11.017896000
Cl	-2.013375000	9.628423000	8.556797000
C	0.447229000	9.290415000	9.688290000
Cl	-0.063636000	10.415236000	10.906005000
O	1.749808000	6.587166000	6.821375000
N	3.251856000	7.818169000	5.098061000
H	3.444388000	7.287929000	4.237124000
H	2.351038000	7.446895000	5.544627000
N	4.270942000	7.525547000	6.085777000
H	4.319888000	8.335354000	6.718247000
H	5.172250000	7.459590000	5.599378000
H	3.193228000	8.822061000	4.871666000

[Mo]-$N_2H_4^-$

Mo	3.565382000	5.666220000	7.396106000
Fe	2.981019000	3.025236000	6.954080000
Fe	4.806795000	3.851476000	9.027736000
Fe	5.559659000	4.176419000	6.229892000
S	3.511450000	4.527627000	5.334252000
S	2.519703000	4.179481000	8.902651000
S	5.841197000	5.584486000	7.983649000
S	5.157132000	2.189850000	7.373697000
Cl	5.551093000	3.403544000	11.125780000
Cl	1.372172000	1.525042000	6.393390000
Cl	7.243998000	4.252216000	4.725577000
C	1.230917000	7.415719000	7.764667000
C	2.139600000	7.813778000	8.802025000
C	-0.038146000	8.012333000	7.697764000
O	3.330229000	7.257019000	8.804264000
Cl	-1.097799000	7.527325000	6.400117000
C	1.724968000	8.785984000	9.730059000
C	-0.440829000	8.983567000	8.638822000
Cl	2.855852000	9.260928000	10.969740000
Cl	-2.028337000	9.689303000	8.536505000
C	0.442357000	9.369236000	9.656772000
Cl	-0.041504000	10.557515000	10.833014000
O	1.665830000	6.521618000	6.897020000
N	3.349776000	7.927039000	5.040154000
H	3.564982000	7.281838000	4.270639000
H	2.412844000	7.618152000	5.347216000
N	4.210036000	7.476725000	6.139482000
H	4.296660000	8.264711000	6.791229000
H	5.146867000	7.329696000	5.752829000

[Mo]-N_2H_5

Mo	3.603198000	5.685729000	7.410959000
Fe	3.114216000	3.115657000	6.982689000

Fe	4.731061000	3.819877000	8.897255000
Fe	5.493970000	4.130512000	6.376595000
S	3.561159000	4.571263000	5.329321000
S	2.478234000	4.251322000	8.863536000
S	5.851613000	5.573474000	8.049061000
S	5.184233000	2.160439000	7.354837000
Cl	5.368357000	3.246909000	10.989982000
Cl	1.534527000	1.580681000	6.460790000
Cl	7.182966000	4.262239000	4.897908000
C	1.268573000	7.502857000	7.728878000
C	2.162253000	7.843745000	8.789793000
C	-0.000834000	8.087854000	7.668300000
O	3.359584000	7.298095000	8.782144000
Cl	-1.032405000	7.667670000	6.329874000
C	1.729767000	8.764735000	9.763549000
C	-0.424530000	9.002903000	8.653881000
Cl	2.840608000	9.181124000	11.036306000
Cl	-2.014690000	9.698133000	8.570019000
C	0.445318000	9.338827000	9.702101000
Cl	-0.063495000	10.457298000	10.931563000
O	1.729518000	6.651308000	6.804981000
N	3.278997000	7.760322000	5.091920000
H	3.439833000	7.125184000	4.296307000
H	2.365672000	7.448607000	5.589916000
N	4.291445000	7.502837000	6.096431000
H	4.334401000	8.328928000	6.706367000
H	5.197065000	7.404448000	5.625127000
H	3.265181000	8.737426000	4.767247000

[Mo]-N_2H_5 (Transition State)

Mo	3.628264000	5.714153000	7.397089000
Fe	3.100520000	3.117605000	7.042748000
Fe	4.754993000	3.795659000	8.927953000
Fe	5.508233000	4.086377000	6.395747000
S	3.565842000	4.523176000	5.360742000
S	2.520885000	4.288590000	8.920476000
S	5.884601000	5.518239000	8.063625000
S	5.143361000	2.116265000	7.383315000
Cl	5.437022000	3.256575000	10.996933000
Cl	1.478595000	1.638203000	6.551245000
Cl	7.208258000	4.191858000	4.946898000
C	1.264834000	7.453089000	7.725633000
C	2.154784000	7.838473000	8.778126000
C	-0.012217000	8.024785000	7.653464000
O	3.360200000	7.310829000	8.776060000
Cl	-1.041121000	7.558823000	6.327146000
C	1.712626000	8.777403000	9.728050000
C	-0.445277000	8.960016000	8.616168000
Cl	2.821289000	9.242835000	10.987286000
Cl	-2.044854000	9.632927000	8.517139000
C	0.420103000	9.334053000	9.654670000
Cl	-0.101625000	10.476197000	10.857390000
O	1.733627000	6.588737000	6.830468000
N	3.202394000	7.912460000	5.004102000
H	3.464067000	7.458873000	4.126118000
H	2.350171000	7.494730000	5.444139000
N	4.363880000	7.397281000	6.216012000
H	4.452615000	8.268218000	6.754619000
H	5.208178000	7.291316000	5.642616000
H	3.181542000	8.932659000	4.921656000

[Mo]-NH_2

Mo	3.491810000	5.962392000	7.132583000
Fe	3.276013000	2.856717000	7.280661000
Fe	4.764966000	4.356336000	8.866421000
Fe	5.385069000	4.139333000	6.321855000
S	3.305017000	4.170625000	5.471711000
S	2.481675000	4.398475000	8.771918000
S	5.778012000	5.926108000	7.647673000
S	5.474723000	2.492293000	7.761344000
Cl	5.400757000	4.344594000	10.986896000
Cl	2.119425000	0.963832000	7.215237000
Cl	6.877099000	4.105145000	4.699349000
C	1.056143000	7.452070000	7.632253000
C	1.946444000	7.827623000	8.682115000
C	-0.257998000	7.950244000	7.638567000
O	3.176568000	7.339380000	8.599929000
Cl	-1.318818000	7.478594000	6.345971000
C	1.507690000	8.674634000	9.709118000
C	-0.691337000	8.802915000	8.670985000
Cl	2.629193000	9.096804000	10.965759000
Cl	-2.320221000	9.402017000	8.664519000
C	0.188395000	9.166264000	9.706538000
Cl	-0.347855000	10.213272000	10.982801000
O	1.545886000	6.650065000	6.710882000
N	3.893039000	7.182418000	5.635276000
H	3.125842000	7.580648000	5.088754000
H	4.777660000	7.252014000	5.131010000

[Mo]-NH_3^+

Mo	3.534313000	5.559902000	7.374745000
Fe	3.209260000	2.894671000	7.147906000
Fe	5.036533000	3.998835000	8.974201000
Fe	5.493098000	4.006160000	6.351270000
S	3.454466000	4.259356000	5.420901000
S	2.771929000	4.049080000	9.049369000
S	5.838490000	5.697954000	7.802011000
S	5.364003000	2.208002000	7.568066000
Cl	5.955532000	3.764619000	10.944243000
Cl	1.795263000	1.263435000	6.802036000
Cl	7.014613000	4.095333000	4.786038000
C	1.127943000	7.165275000	7.716051000
C	2.058513000	7.725453000	8.645420000
C	-0.164822000	7.707855000	7.603531000
O	3.245063000	7.162096000	8.671157000
Cl	-1.256498000	7.003569000	6.459874000
C	1.679218000	8.817120000	9.447027000
C	-0.535600000	8.793178000	8.410643000
Cl	2.839161000	9.467136000	10.554986000
Cl	-2.126058000	9.457406000	8.278389000
C	0.386083000	9.347390000	9.332099000
Cl	-0.083471000	10.685823000	10.320344000
O	1.574278000	6.155518000	7.003988000
N	3.937219000	7.271614000	5.930319000
H	3.105492000	7.460703000	5.362109000
H	4.153551000	8.130206000	6.446775000
H	4.712864000	7.107122000	5.282926000

[Mo]-NH_2^-

Mo	3.497070000	5.732831000	7.176511000
Fe	3.191626000	3.031034000	7.252744000
Fe	4.939769000	4.195209000	8.816492000
Fe	5.406091000	4.039831000	6.211055000
S	3.322190000	4.222907000	5.365037000
S	2.687320000	4.396521000	9.002529000
S	5.824668000	5.809176000	7.561400000
S	5.344907000	2.306019000	7.570274000
Cl	5.864919000	4.052849000	10.863840000
Cl	1.704979000	1.339403000	7.116607000
Cl	6.900254000	3.959899000	4.559157000
C	1.046364000	7.247254000	7.661204000
C	1.980084000	7.798453000	8.608689000

C	-0.276068000	7.719031000	7.647564000
O	3.203108000	7.338749000	8.586672000
Cl	-1.376407000	7.024336000	6.485072000
C	1.532289000	8.804028000	9.487311000
C	-0.707473000	8.725132000	8.540302000
Cl	2.688001000	9.466907000	10.613750000
Cl	-2.357399000	9.279695000	8.497057000
C	0.199765000	9.268200000	9.458765000
Cl	-0.312835000	10.509229000	10.568018000
O	1.499891000	6.324424000	6.838293000
N	3.843987000	7.332631000	5.852246000
H	3.005486000	7.923146000	5.826607000
H	4.612607000	7.937918000	6.152938000

[Mo]-NH_3

Mo	3.553698000	5.592364000	7.335309000
Fe	3.228008000	2.960077000	7.212715000
Fe	4.948520000	3.997751000	8.937785000
Fe	5.511784000	3.980035000	6.351970000
S	3.475748000	4.239514000	5.406699000
S	2.685819000	4.218880000	9.028291000
S	5.863996000	5.680536000	7.799467000
S	5.347802000	2.186530000	7.598464000
Cl	5.777477000	3.753415000	10.990836000
Cl	1.746931000	1.319365000	6.943823000
Cl	7.063452000	4.005212000	4.774483000
C	1.153835000	7.205736000	7.666402000
C	2.083836000	7.765628000	8.595670000
C	-0.158418000	7.699187000	7.622805000
O	3.309330000	7.270832000	8.573707000
Cl	-1.258865000	6.998187000	6.470608000
C	1.668247000	8.800324000	9.446712000
C	-0.567581000	8.737370000	8.485557000
Cl	2.832875000	9.465051000	10.556410000
Cl	-2.202006000	9.323582000	8.420219000
C	0.345700000	9.287954000	9.396936000
Cl	-0.147307000	10.563360000	10.469361000
O	1.613361000	6.250150000	6.877197000
N	4.019510000	7.274600000	5.852190000
H	3.185186000	7.466886000	5.290312000
H	4.247046000	8.126504000	6.373081000
H	4.792459000	7.085785000	5.209410000

$[V]^{2+}$-Acetonitrile

V	3.277727000	5.084573000	7.748330000
Fe	2.945342000	2.331378000	7.668184000
Fe	4.970910000	3.564852000	9.334714000
Fe	5.279584000	3.491638000	6.492189000
S	3.074258000	3.697174000	5.886818000
S	2.681221000	3.689679000	9.504074000
S	5.594395000	5.229938000	7.958913000
S	5.219237000	1.720404000	7.877427000
Cl	6.100453000	3.401735000	11.297419000
Cl	1.491993000	0.595921000	7.499842000
Cl	6.677945000	3.564334000	4.727810000
C	0.927233000	6.643654000	8.193021000
C	1.933599000	7.255773000	9.025991000
C	-0.371651000	7.183043000	8.189612000
O	3.124842000	6.722403000	8.981719000
Cl	-1.564453000	6.426238000	7.160097000
C	1.580252000	8.367468000	9.811980000
C	-0.706708000	8.303738000	8.983675000
Cl	2.822385000	9.086475000	10.809828000
Cl	-2.329029000	8.940770000	8.950693000
C	0.268805000	8.895337000	9.795181000
Cl	-0.127375000	10.275524000	10.783676000
O	1.309319000	5.616369000	7.482996000
C	3.780120000	8.569201000	4.517520000
H	4.028916000	9.514125000	5.020358000
H	2.839307000	8.696036000	3.963977000
H	4.581360000	8.320807000	3.807393000
C	3.641027000	7.511008000	5.493295000
N	3.536430000	6.655759000	6.268463000

$[V]^{2+}$-N_2H_4

V	3.565908000	5.861589000	7.313720000
Fe	2.907541000	3.187059000	6.896464000
Fe	4.782252000	4.049165000	9.042276000
Fe	5.567737000	3.873284000	6.360752000
S	3.655187000	4.622374000	5.321722000
S	2.516868000	4.395642000	8.817479000
S	5.843191000	5.631346000	7.828500000
S	4.893367000	2.168583000	7.633470000
Cl	5.661706000	3.706638000	11.123185000
Cl	1.319542000	1.713264000	6.169887000
Cl	7.307180000	3.551931000	4.959285000
C	1.245811000	7.448614000	7.729833000
C	2.161210000	7.856287000	8.763593000
C	-0.066968000	7.942434000	7.741625000
O	3.380393000	7.388237000	8.668085000
Cl	-1.135699000	7.450863000	6.453179000
C	1.709265000	8.734242000	9.763381000
C	-0.506609000	8.820919000	8.757259000
Cl	2.845838000	9.223383000	10.993293000
Cl	-2.149752000	9.398265000	8.754694000
C	0.381035000	9.215034000	9.766905000
Cl	-0.151018000	10.286389000	11.033090000
O	1.741022000	6.653053000	6.807602000
N	3.417286000	8.013348000	4.957508000
H	3.580742000	7.343307000	4.196230000
H	2.474239000	7.756024000	5.293600000
N	4.275831000	7.532924000	6.046326000
H	4.417400000	8.324794000	6.683107000
H	5.193398000	7.325092000	5.643067000

$[V]^{2+}$-$N_2H_5^+$

V	3.602335000	5.736740000	7.375274000
Fe	3.070149000	3.183713000	6.995329000
Fe	4.697216000	3.917723000	8.901891000
Fe	5.490426000	4.075529000	6.381779000
S	3.555495000	4.601233000	5.342011000
S	2.467542000	4.390897000	8.827248000
S	5.818510000	5.620339000	7.993432000
S	5.092171000	2.183452000	7.441502000
Cl	5.336022000	3.398527000	11.007638000
Cl	1.471144000	1.666492000	6.486366000
Cl	7.178521000	4.173858000	4.911010000
C	1.289479000	7.470069000	7.689527000
C	2.195655000	7.826808000	8.739349000
C	0.001235000	8.013103000	7.663084000
O	3.398583000	7.313216000	8.691959000
Cl	-1.045035000	7.577964000	6.338203000
C	1.749907000	8.719531000	9.734682000
C	-0.433721000	8.900485000	8.671210000
Cl	2.871498000	9.155674000	10.994027000
Cl	-2.050344000	9.541341000	8.629589000
C	0.444806000	9.250519000	9.706203000
Cl	-0.078011000	10.332982000	10.964493000
O	1.770023000	6.644883000	6.757825000
N	3.293782000	7.774181000	5.092279000
H	3.450346000	7.171502000	4.273620000

H	2.384893000	7.426524000	5.609401000
N	4.312243000	7.483649000	6.080017000
H	4.360336000	8.285809000	6.719113000
H	5.214061000	7.392231000	5.602333000
H	3.270628000	8.760890000	4.801625000

[V]$^{2+}$-$N_2H_4^-$

V	3.564779000	5.714701000	7.363936000
Fe	3.103400000	3.140193000	7.010390000
Fe	4.723000000	3.881781000	8.867340000
Fe	5.449820000	4.145712000	6.349211000
S	3.494015000	4.583344000	5.328274000
S	2.466770000	4.306758000	8.877391000
S	5.802979000	5.650916000	7.984465000
S	5.181746000	2.160476000	7.364536000
Cl	5.472562000	3.313580000	11.010319000
Cl	1.507945000	1.511202000	6.491775000
Cl	7.162746000	4.297055000	4.820940000
C	1.240096000	7.426940000	7.732524000
C	2.162323000	7.831368000	8.776788000
C	-0.042257000	8.004445000	7.702996000
O	3.349136000	7.304640000	8.753557000
Cl	-1.115026000	7.527663000	6.402679000
C	1.726150000	8.785552000	9.719308000
C	-0.458906000	8.953954000	8.665577000
Cl	2.867915000	9.285942000	10.950082000
Cl	-2.072720000	9.616253000	8.602103000
C	0.427878000	9.344267000	9.674620000
Cl	-0.069887000	10.500847000	10.884321000
O	1.684400000	6.570804000	6.853240000
N	3.394146000	7.911556000	5.030862000
H	3.544237000	7.210000000	4.293636000
H	2.456137000	7.646772000	5.382257000
N	4.250554000	7.429538000	6.124954000
H	4.392052000	8.214623000	6.769152000
H	5.167913000	7.203200000	5.731896000

[V]$^{2+}$-N_2H_5

V	3.621056000	5.751396000	7.360253000
Fe	3.090714000	3.195724000	6.991146000
Fe	4.684438000	3.915490000	8.883129000
Fe	5.477921000	4.151899000	6.378492000
S	3.548082000	4.626090000	5.309801000
S	2.439324000	4.408628000	8.816229000
S	5.831093000	5.648205000	8.015384000
S	5.138171000	2.183540000	7.402614000
Cl	5.292880000	3.344085000	11.048601000
Cl	1.477898000	1.613666000	6.480537000
Cl	7.187407000	4.255986000	4.850395000
C	1.278935000	7.523297000	7.683700000
C	2.190024000	7.862779000	8.745977000
C	-0.013979000	8.058029000	7.680027000
O	3.391445000	7.367703000	8.694980000
Cl	-1.065006000	7.647044000	6.345570000
C	1.727661000	8.731251000	9.759650000
C	-0.460734000	8.916486000	8.709990000
Cl	2.847177000	9.155593000	11.030582000
Cl	-2.088533000	9.536165000	8.690325000
C	0.416738000	9.250384000	9.749393000
Cl	-0.116400000	10.296142000	11.038058000
O	1.752358000	6.725586000	6.733580000
N	3.336359000	7.700756000	5.056159000
H	3.441360000	6.941598000	4.360193000
H	2.407402000	7.460041000	5.618577000
N	4.337817000	7.466968000	6.073373000
H	4.378083000	8.297255000	6.675254000
H	5.247445000	7.328655000	5.623094000
H	3.382758000	8.629422000	4.616958000

[V]$^{2+}$-N_2H_5 (Transition State)

V	3.604415000	5.733833000	7.371709000
Fe	3.089407000	3.158710000	7.046681000
Fe	4.738659000	3.870979000	8.887691000
Fe	5.465675000	4.119488000	6.351738000
S	3.503782000	4.571232000	5.357712000
S	2.499986000	4.368300000	8.897164000
S	5.833402000	5.604413000	7.985126000
S	5.145551000	2.140628000	7.381428000
Cl	5.480760000	3.330935000	10.998785000
Cl	1.445969000	1.606786000	6.576258000
Cl	7.167175000	4.252874000	4.839561000
C	1.271077000	7.458062000	7.702079000
C	2.173844000	7.831150000	8.764682000
C	-0.011877000	8.022297000	7.659767000
O	3.369372000	7.318332000	8.742780000
Cl	-1.051401000	7.581221000	6.324395000
C	1.713273000	8.745036000	9.736556000
C	-0.454683000	8.931975000	8.647038000
Cl	2.824854000	9.203196000	11.004695000
Cl	-2.068797000	9.585147000	8.575244000
C	0.412209000	9.290121000	9.686500000
Cl	-0.118422000	10.395695000	10.926084000
O	1.740510000	6.622900000	6.795901000
N	3.238377000	7.842704000	5.005230000
H	3.465400000	7.291297000	4.174691000
H	2.382683000	7.472113000	5.504364000
N	4.376096000	7.350370000	6.222623000
H	4.466653000	8.220278000	6.762907000
H	5.229854000	7.219674000	5.669638000
H	3.246088000	8.850119000	4.822990000

[V]$^{2+}$-NH_2

V	3.617819000	6.042556000	7.207640000
Fe	3.000937000	3.426463000	6.935449000
Fe	4.767584000	3.688272000	8.867409000
Fe	5.456296000	4.172436000	6.358633000
S	3.578305000	4.794506000	5.272488000
S	2.652571000	4.528328000	8.911056000
S	5.801779000	5.507593000	8.130707000
S	4.920343000	2.173089000	7.146603000
Cl	5.468011000	2.742245000	10.802673000
Cl	1.223992000	2.119331000	6.452106000
Cl	7.198242000	4.267373000	4.951174000
C	1.232679000	7.423881000	7.756043000
C	2.170919000	7.988784000	8.688533000
C	-0.109472000	7.830943000	7.789892000
O	3.399963000	7.563532000	8.595675000
Cl	-1.209285000	7.123172000	6.636952000
C	1.709867000	8.943623000	9.615090000
C	-0.553836000	8.789324000	8.726571000
Cl	2.866807000	9.617525000	10.734440000
Cl	-2.226268000	9.273468000	8.749819000
C	0.357202000	9.343521000	9.638040000
Cl	-0.179179000	10.522266000	10.802155000
O	1.723965000	6.550425000	6.906964000
N	4.506697000	7.444493000	6.242125000
H	4.793896000	8.264425000	6.783490000
H	5.173269000	7.268396000	5.488331000

[V]$^{2+}$-NH_3^+

V	3.518796000	5.773684000	7.391278000
Fe	3.067297000	3.220618000	6.938303000

Fe	4.911598000	3.723692000	8.856924000
Fe	5.429311000	4.166958000	6.291679000
S	3.462385000	4.712731000	5.352642000
S	2.695288000	4.240838000	8.944544000
S	5.789847000	5.584855000	8.003275000
S	5.101333000	2.186141000	7.173542000
Cl	5.726274000	2.976294000	10.783404000
Cl	1.404772000	1.806685000	6.463785000
Cl	7.048519000	4.378381000	4.790406000
C	1.261611000	7.411745000	7.770001000
C	2.152680000	7.835436000	8.808522000
C	-0.011684000	7.989216000	7.650253000
O	3.317949000	7.251355000	8.827585000
Cl	-1.050335000	7.440936000	6.369117000
C	1.725524000	8.837305000	9.702985000
C	-0.425385000	8.985670000	8.553618000
Cl	2.826247000	9.342401000	10.950895000
Cl	-2.005900000	9.688884000	8.405446000
C	0.446026000	9.407565000	9.577196000
Cl	-0.061596000	10.637885000	10.692168000
O	1.737674000	6.477326000	6.971608000
N	4.278756000	7.525402000	6.271170000
H	4.584938000	8.238155000	6.939148000
H	5.059121000	7.323337000	5.642155000
H	3.524202000	7.928222000	5.708920000

[V]$^{2+}$-NH_2^-

V	3.591371000	5.834571000	7.309414000
Fe	3.096418000	3.240049000	6.902143000
Fe	4.726345000	3.872278000	8.797476000
Fe	5.503489000	4.174040000	6.291542000
S	3.554446000	4.705043000	5.281553000
S	2.499424000	4.308155000	8.819965000
S	5.819352000	5.634151000	7.978831000
S	5.139232000	2.198966000	7.250494000
Cl	5.483751000	3.262715000	10.891538000
Cl	1.493679000	1.701426000	6.285114000
Cl	7.213697000	4.320933000	4.806871000
C	1.219376000	7.401790000	7.760594000
C	2.153311000	7.846200000	8.781118000
C	-0.068010000	7.978833000	7.737748000
O	3.334889000	7.301501000	8.772496000
Cl	-1.173993000	7.435359000	6.492415000
C	1.733043000	8.828549000	9.698055000
C	-0.465059000	8.966197000	8.670010000
Cl	2.885817000	9.350314000	10.910580000
Cl	-2.074616000	9.639738000	8.597990000
C	0.435162000	9.392592000	9.651258000
Cl	-0.035974000	10.604706000	10.816272000
O	1.639393000	6.499378000	6.932682000
N	4.140498000	7.527374000	6.198317000
H	4.546594000	8.127128000	6.929739000
H	4.951468000	7.257984000	5.625844000

[V]$^{2+}$-NH_3

V	3.594059000	5.763197000	7.370759000
Fe	3.087334000	3.204405000	6.922865000
Fe	4.729172000	3.872169000	8.839327000
Fe	5.492818000	4.125359000	6.314670000
S	3.548625000	4.678017000	5.316739000
S	2.495331000	4.317849000	8.818665000
S	5.830050000	5.610097000	7.980817000
S	5.116885000	2.185649000	7.314356000
Cl	5.423211000	3.289758000	10.932864000
Cl	1.484498000	1.690073000	6.340842000
Cl	7.180054000	4.256904000	4.826261000
C	1.294697000	7.434432000	7.726541000
C	2.194812000	7.823409000	8.787318000
C	0.013137000	8.011038000	7.680171000
O	3.379618000	7.272393000	8.777257000
Cl	-1.047333000	7.530597000	6.375827000
C	1.760320000	8.772151000	9.730030000
C	-0.406857000	8.958821000	8.641350000
Cl	2.883161000	9.245140000	10.984030000
Cl	-2.011046000	9.636016000	8.554141000
C	0.467261000	9.340621000	9.665654000
Cl	-0.036206000	10.500464000	10.865963000
O	1.755337000	6.574812000	6.856739000
N	4.258458000	7.525890000	6.184422000
H	4.503669000	8.272338000	6.839573000
H	5.055496000	7.358741000	5.567480000
H	3.468685000	7.841377000	5.615908000

[V]$^{3+}$-Acetonitrile

V	3.233830000	5.380349000	7.578993000
Fe	2.769933000	2.614289000	7.631445000
Fe	4.823142000	3.859659000	9.321778000
Fe	5.273638000	3.384234000	6.575525000
S	3.191268000	3.805506000	5.781754000
S	2.540435000	4.094577000	9.370802000
S	5.613555000	5.278020000	7.778520000
S	4.919713000	1.850814000	8.134934000
Cl	5.885211000	3.820385000	11.279521000
Cl	1.235347000	1.012369000	7.444689000
Cl	6.800914000	2.948296000	5.048737000
C	0.890450000	6.734297000	8.173483000
C	1.894664000	7.349212000	8.996768000
C	-0.460503000	7.052650000	8.380076000
O	3.134785000	7.020752000	8.709379000
Cl	-1.646632000	6.285625000	7.364948000
C	1.508442000	8.262296000	9.989867000
C	-0.835262000	7.964372000	9.387208000
Cl	2.754290000	8.990194000	10.961747000
Cl	-2.514881000	8.332202000	9.637250000
C	0.147429000	8.568837000	10.190060000
Cl	-0.311232000	9.688621000	11.436858000
O	1.345281000	5.928167000	7.239794000
C	4.157616000	8.511668000	4.115931000
H	4.426440000	9.482143000	4.555550000
H	3.267513000	8.636516000	3.483782000
H	4.991606000	8.152093000	3.497174000
C	3.882555000	7.558971000	5.165468000
N	3.667895000	6.788242000	6.003154000

[V]$^{3+}$-N_2H_4

V	3.545453000	5.928321000	7.300974000
Fe	2.861520000	3.257962000	6.936907000
Fe	4.711136000	4.116962000	9.055910000
Fe	5.565842000	3.893776000	6.357843000
S	3.669107000	4.602257000	5.325754000
S	2.467676000	4.538493000	8.803587000
S	5.846793000	5.615719000	7.820571000
S	4.843919000	2.248769000	7.654629000
Cl	5.460406000	3.777492000	11.125658000
Cl	1.255630000	1.838838000	6.328715000
Cl	7.275267000	3.462815000	5.038727000
C	1.266090000	7.466532000	7.710516000
C	2.180712000	7.872790000	8.737348000
C	-0.065273000	7.900089000	7.756508000
O	3.420835000	7.456107000	8.586804000
Cl	-1.137026000	7.397124000	6.483216000
C	1.725235000	8.693005000	9.780368000

C	-0.513225000	8.719801000	8.813029000
Cl	2.861265000	9.164684000	11.009125000
Cl	-2.172402000	9.231318000	8.864639000
C	0.380255000	9.114088000	9.823013000
Cl	-0.169530000	10.114559000	11.132308000
O	1.789356000	6.725060000	6.750491000
N	3.501607000	8.012483000	4.867803000
H	3.662378000	7.338402000	4.110688000
H	2.541685000	7.804231000	5.183399000
N	4.329499000	7.536734000	5.976690000
H	4.481668000	8.340725000	6.595759000
H	5.248023000	7.304946000	5.588139000

[V]$^{3+}$-$N_2H_5^+$

V	3.584826000	5.733604000	7.389342000
Fe	3.140531000	3.144401000	7.049168000
Fe	4.683265000	3.862406000	8.859387000
Fe	5.469767000	4.127454000	6.371467000
S	3.518600000	4.565720000	5.392766000
S	2.508255000	4.325967000	8.815903000
S	5.784543000	5.595738000	8.018041000
S	5.126697000	2.214229000	7.428392000
Cl	5.248274000	3.370150000	10.925542000
Cl	1.549420000	1.684741000	6.647657000
Cl	7.110004000	4.218457000	4.912350000
C	1.296541000	7.436288000	7.699663000
C	2.189032000	7.788084000	8.751242000
C	0.019819000	8.003129000	7.641126000
O	3.384947000	7.233681000	8.717642000
Cl	-1.014525000	7.567278000	6.313180000
C	1.763988000	8.702700000	9.730559000
C	-0.401697000	8.914992000	8.631161000
Cl	2.878760000	9.125038000	10.994463000
Cl	-1.996996000	9.595082000	8.557948000
C	0.471973000	9.261684000	9.674362000
Cl	-0.039936000	10.375681000	10.904173000
O	1.782110000	6.583841000	6.789437000
N	3.267820000	7.808933000	5.104340000
H	3.464360000	7.307174000	4.228710000
H	2.371848000	7.408942000	5.561421000
N	4.288484000	7.497511000	6.084390000
H	4.344640000	8.296305000	6.728282000
H	5.186970000	7.423810000	5.595579000
H	3.197576000	8.816844000	4.904328000

[V]$^{3+}$-$N_2H_4^-$

V	3.545363000	5.710398000	7.388045000
Fe	3.091548000	3.135281000	7.003554000
Fe	4.740026000	3.888305000	8.882696000
Fe	5.465386000	4.103686000	6.346420000
S	3.495102000	4.583633000	5.359282000
S	2.494654000	4.284237000	8.878670000
S	5.789713000	5.634313000	7.973063000
S	5.153179000	2.178814000	7.391835000
Cl	5.464686000	3.374281000	10.980652000
Cl	1.518057000	1.570692000	6.488393000
Cl	7.118841000	4.264850000	4.827149000
C	1.249572000	7.397199000	7.744823000
C	2.165175000	7.802719000	8.781120000
C	-0.026595000	7.980526000	7.693756000
O	3.350166000	7.252924000	8.763603000
Cl	-1.094265000	7.489596000	6.400310000
C	1.743488000	8.768999000	9.713204000
C	-0.434186000	8.945730000	8.642116000
Cl	2.882604000	9.261669000	10.943016000
Cl	-2.033872000	9.630587000	8.556535000
C	0.452522000	9.339035000	9.652114000
Cl	-0.035516000	10.519054000	10.838101000
O	1.698033000	6.514859000	6.882016000
N	3.341453000	7.948958000	5.078285000
H	3.584037000	7.384214000	4.256829000
H	2.418674000	7.578568000	5.362566000
N	4.215043000	7.449177000	6.145051000
H	4.346668000	8.218578000	6.810200000
H	5.134348000	7.263202000	5.735728000

[V]$^{3+}$-N_2H_5

V	3.606010000	5.740167000	7.375593000
Fe	3.055136000	3.192048000	6.986026000
Fe	4.690683000	3.918237000	8.908892000
Fe	5.483535000	4.066696000	6.390426000
S	3.560970000	4.611880000	5.339856000
S	2.461877000	4.399335000	8.823570000
S	5.821622000	5.615798000	7.997331000
S	5.065258000	2.179380000	7.453948000
Cl	5.321766000	3.415301000	11.020115000
Cl	1.442009000	1.698254000	6.460446000
Cl	7.177900000	4.118806000	4.923965000
C	1.296806000	7.478408000	7.691870000
C	2.203035000	7.832246000	8.741195000
C	0.008576000	8.021589000	7.665057000
O	3.404910000	7.315626000	8.693211000
Cl	-1.039001000	7.588439000	6.341576000
C	1.760112000	8.725774000	9.736937000
C	-0.424735000	8.909280000	8.674144000
Cl	2.882430000	9.161037000	10.995255000
Cl	-2.040077000	9.551853000	8.634467000
C	0.455386000	9.258128000	9.708752000
Cl	-0.065782000	10.341199000	10.966887000
O	1.776687000	6.654283000	6.758076000
N	3.303496000	7.797406000	5.106774000
H	3.455034000	7.211162000	4.275570000
H	2.395622000	7.442784000	5.622532000
N	4.325401000	7.488145000	6.085589000
H	4.383555000	8.282585000	6.733489000
H	5.223493000	7.395090000	5.601547000
H	3.284080000	8.789469000	4.834539000

[V]$^{3+}$-N_2H_5(TS)

V	3.507770000	5.678245000	7.388552000
Fe	3.085578000	3.088752000	7.073852000
Fe	4.859950000	3.831051000	8.904723000
Fe	5.424997000	4.027985000	6.319591000
S	3.425008000	4.514081000	5.399294000
S	2.617916000	4.256105000	8.977900000
S	5.826666000	5.551451000	7.944910000
S	5.141018000	2.133570000	7.389200000
Cl	5.660684000	3.282897000	10.916186000
Cl	1.475488000	1.573484000	6.672657000
Cl	7.015262000	4.180022000	4.768663000
C	1.246968000	7.365308000	7.718443000
C	2.149729000	7.788604000	8.748068000
C	-0.020290000	7.954349000	7.614781000
O	3.325033000	7.216618000	8.763718000
Cl	-1.065178000	7.429161000	6.322688000
C	1.727502000	8.788571000	9.645700000
C	-0.430565000	8.952131000	8.524161000
Cl	2.846162000	9.298233000	10.880451000
Cl	-2.014751000	9.655604000	8.386877000
C	0.446018000	9.365936000	9.538993000
Cl	-0.049043000	10.588799000	10.672569000
O	1.709249000	6.444785000	6.886170000

N	3.178937000	8.158841000	5.137260000
H	3.491540000	8.012824000	4.178060000
H	2.366805000	7.587568000	5.402386000
N	4.422750000	7.215502000	6.374041000
H	4.655381000	8.043498000	6.936845000
H	5.165677000	7.071422000	5.681122000
H	3.083533000	9.145007000	5.379774000

[V]$^{3+}$-NH$_2$

V	3.489381000	6.033272000	7.108889000
Fe	3.296487000	2.930991000	7.350414000
Fe	4.754892000	4.349805000	8.838227000
Fe	5.334104000	4.203279000	6.296347000
S	3.229267000	4.214200000	5.554091000
S	2.526162000	4.434849000	8.759001000
S	5.719615000	5.949418000	7.642990000
S	5.469409000	2.538203000	7.746228000
Cl	5.366826000	4.359804000	10.955607000
Cl	2.127907000	1.073435000	7.464627000
Cl	6.773732000	4.174244000	4.630975000
C	1.092494000	7.438423000	7.600443000
C	1.962060000	7.777733000	8.683837000
C	-0.216884000	7.951771000	7.585880000
O	3.175734000	7.266925000	8.615255000
Cl	-1.251466000	7.523761000	6.254993000
C	1.501849000	8.601564000	9.721634000
C	-0.668048000	8.784405000	8.627481000
Cl	2.591335000	8.971185000	11.024576000
Cl	-2.288977000	9.407675000	8.592769000
C	0.188455000	9.109561000	9.695165000
Cl	-0.369742000	10.132439000	10.983104000
O	1.605383000	6.649463000	6.688618000
N	3.912343000	7.166569000	5.657873000
H	3.153028000	7.568943000	5.104193000
H	4.789851000	7.172895000	5.136911000

[V]$^{3+}$-NH$_3^+$

V	3.537826000	5.651068000	7.311653000
Fe	3.272710000	2.979501000	7.259651000
Fe	4.935423000	3.934637000	8.904236000
Fe	5.460457000	4.071154000	6.331161000
S	3.416638000	4.280343000	5.477568000
S	2.756660000	4.227445000	8.998160000
S	5.779548000	5.683338000	7.837093000
S	5.354046000	2.227363000	7.528465000
Cl	5.717494000	3.588776000	10.907493000
Cl	1.823665000	1.358091000	7.108273000
Cl	6.969738000	4.171004000	4.753879000
C	1.160513000	7.160873000	7.676451000
C	2.096297000	7.736391000	8.606636000
C	-0.155414000	7.665925000	7.595711000
O	3.281892000	7.208354000	8.597942000
Cl	-1.245090000	6.945770000	6.462553000
C	1.691057000	8.805404000	9.436476000
C	-0.540725000	8.722876000	8.425988000
Cl	2.843901000	9.471116000	10.540227000
Cl	-2.149614000	9.343957000	8.336193000
C	0.383643000	9.292490000	9.346460000
Cl	-0.117335000	10.597781000	10.360180000
O	1.629421000	6.187334000	6.953435000
N	4.003255000	7.269448000	5.907865000
H	3.174023000	7.515964000	5.359202000
H	4.290398000	8.108754000	6.419833000
H	4.750010000	7.049655000	5.243639000

[V]$^{3+}$-NH$_2^-$

V	3.485979000	6.005396000	7.161517000
Fe	3.262474000	2.915264000	7.269480000
Fe	4.761817000	4.354431000	8.878957000
Fe	5.387722000	4.182896000	6.331857000
S	3.303871000	4.245822000	5.494827000
S	2.476585000	4.424780000	8.778153000
S	5.730537000	5.962495000	7.678461000
S	5.468641000	2.481754000	7.744555000
Cl	5.444853000	4.299893000	11.032343000
Cl	2.094663000	0.976624000	7.153773000
Cl	6.898635000	4.193023000	4.675658000
C	1.070761000	7.427112000	7.625075000
C	1.965580000	7.817370000	8.681765000
C	-0.244758000	7.928249000	7.634732000
O	3.183320000	7.329280000	8.610611000
Cl	-1.311208000	7.441291000	6.342062000
C	1.511942000	8.680441000	9.690622000
C	-0.686522000	8.797059000	8.654349000
Cl	2.631729000	9.129896000	10.949937000
Cl	-2.321530000	9.395720000	8.634308000
C	0.189309000	9.174853000	9.682736000
Cl	-0.348263000	10.243777000	10.947458000
O	1.562592000	6.625344000	6.721766000
N	3.888144000	7.180428000	5.697193000
H	3.107140000	7.450680000	5.093595000
H	4.751185000	7.120934000	5.154340000

[V]$^{3+}$-NH$_3$

V	3.489744000	5.632208000	7.334398000
Fe	3.178137000	3.019468000	7.222458000
Fe	5.082023000	3.903865000	8.968580000
Fe	5.438767000	4.026463000	6.333432000
S	3.394886000	4.322763000	5.446827000
S	2.838372000	4.251818000	9.109438000
S	5.789239000	5.675949000	7.823790000
S	5.291782000	2.166918000	7.490395000
Cl	6.064201000	3.497333000	10.918597000
Cl	1.607562000	1.449145000	7.002366000
Cl	6.968708000	4.134519000	4.730588000
C	1.154447000	7.167208000	7.682108000
C	2.081248000	7.764424000	8.595511000
C	-0.161878000	7.647644000	7.595734000
O	3.283530000	7.259861000	8.592954000
Cl	-1.244320000	6.890021000	6.466410000
C	1.647468000	8.835685000	9.402317000
C	-0.581873000	8.714467000	8.411279000
Cl	2.793205000	9.548710000	10.498720000
Cl	-2.215115000	9.294539000	8.308573000
C	0.325727000	9.306657000	9.312119000
Cl	-0.190114000	10.623926000	10.318746000
O	1.640154000	6.178267000	6.959959000
N	4.052181000	7.271681000	5.954437000
H	3.233192000	7.562215000	5.413403000
H	4.366905000	8.076861000	6.502560000
H	4.792258000	7.032198000	5.290722000

[Mo]-ACN (M_S = 8.5)

Mo	2.969148000	5.736443000	7.634922000
Fe	3.143008000	2.374460000	7.493259000
Fe	5.086270000	3.560848000	9.095090000
Fe	5.309801000	3.969493000	6.103133000
S	3.013619000	3.960869000	5.776051000
S	2.788763000	3.823203000	9.269294000
S	5.518780000	5.490702000	7.841630000
S	5.494105000	2.023873000	7.319530000
Cl	6.230657000	3.067198000	10.930220000

Cl	1.990345000	0.479765000	7.439033000
Cl	6.530594000	4.445738000	4.271418000
C	0.501187000	6.928769000	8.500111000
C	1.505601000	7.540491000	9.327177000
C	-0.842170000	7.307553000	8.658860000
O	2.752445000	7.158170000	9.125686000
Cl	-2.033758000	6.556771000	7.632411000
C	1.120583000	8.502522000	10.275607000
C	-1.212300000	8.274786000	9.616926000
Cl	2.363257000	9.233746000	11.254504000
Cl	-2.888842000	8.726854000	9.792626000
C	-0.233295000	8.870761000	10.423437000
Cl	-0.683536000	10.069083000	11.609753000
O	0.920231000	6.042017000	7.617359000
C	4.793909000	7.652756000	3.972444000
H	5.411608000	8.557303000	4.078051000
H	4.174625000	7.753071000	3.068473000
H	5.456269000	6.772244000	3.859360000
C	3.968941000	7.446303000	5.144695000
N	3.437598000	7.029567000	6.104138000

[Mo]-N_2H_4 – Fe1Fe2 Bridging

Mo	3.254811000	5.072903000	7.774345000
Fe	3.055518000	2.323684000	7.762999000
Fe	4.837752000	3.438769000	9.306434000
Fe	5.257878000	3.665297000	6.430942000
S	3.036026000	3.690339000	5.892394000
S	2.561842000	3.746904000	9.537795000
S	5.578459000	5.237232000	8.056403000
S	5.398522000	1.814271000	7.588751000
Cl	6.155278000	3.782232000	11.191125000
Cl	1.273160000	0.942043000	7.148527000
Cl	6.547974000	4.062239000	4.639928000
C	0.887135000	6.741534000	8.148164000
C	1.874788000	7.344383000	8.984130000
C	-0.401640000	7.293875000	8.095944000
O	3.072475000	6.784944000	8.981065000
Cl	-1.575045000	6.534683000	7.058591000
C	1.539551000	8.478581000	9.738896000
C	-0.729762000	8.432117000	8.860179000
Cl	2.773752000	9.189113000	10.739257000
Cl	-2.333984000	9.094227000	8.781646000
C	0.241057000	9.024648000	9.681970000
Cl	-0.150459000	10.427006000	10.629835000
O	1.266780000	5.683331000	7.454069000
C	3.777346000	8.629102000	4.516969000
H	4.083859000	9.550883000	5.029365000
H	2.809972000	8.792609000	4.022794000
H	4.530314000	8.366857000	3.761386000
C	3.656584000	7.555637000	5.475925000
N	3.562128000	6.690469000	6.239681000
H	2.382842000	1.065432000	10.094094000
N	3.232220000	0.825962000	9.578371000
H	3.262383000	-0.196653000	9.498427000
H	4.229070000	1.161833000	11.344596000
H	5.183075000	0.804176000	10.041944000
N	4.357697000	1.328334000	10.340272000

[Mo]-N_2H_4 – Fe1 Terminal

Mo	3.251411000	5.038851000	7.653235000
Fe	2.707188000	2.367978000	7.716469000
Fe	4.892366000	3.566174000	9.310463000
Fe	5.159778000	3.318139000	6.465856000
S	2.991850000	3.580957000	5.814092000
S	2.630780000	3.767613000	9.529052000
S	5.604942000	5.129544000	7.841415000
S	5.049947000	1.693651000	7.949129000
Cl	5.995225000	3.509791000	11.254739000
Cl	2.002945000	0.120588000	7.799349000
Cl	6.574795000	3.185934000	4.743525000
C	0.819250000	6.541986000	8.214283000
C	1.825966000	7.197277000	8.983501000
C	-0.519615000	6.934026000	8.340693000
O	3.068695000	6.773008000	8.825634000
Cl	-1.708846000	6.082506000	7.398900000
C	1.455048000	8.243141000	9.843460000
C	-0.882872000	7.980204000	9.212162000
Cl	2.707382000	9.028603000	10.760246000
Cl	-2.549501000	8.444300000	9.359431000
C	0.106541000	8.636550000	9.960694000
Cl	-0.326200000	9.927491000	11.038768000
O	1.230707000	5.578885000	7.399858000
C	3.707278000	8.518418000	4.358918000
H	4.006758000	9.443389000	4.870302000
H	2.735592000	8.671638000	3.869742000
H	4.459432000	8.262532000	3.600182000
C	3.601840000	7.445537000	5.319572000
N	3.517806000	6.582322000	6.086962000
N	0.535299000	2.748965000	7.450106000
H	0.188397000	2.304603000	6.595129000
H	0.425557000	3.767772000	7.324238000
H	-0.075557000	1.380263000	8.735465000
N	-0.369805000	2.345208000	8.526121000
H	-0.043825000	2.880203000	9.341250000

[Mo]-N_2H_4 – Fe2 Terminal

Mo	7.334680000	5.804365000	8.814745000
Fe	5.598891000	3.843518000	9.683091000
Fe	8.363351000	3.284434000	8.794304000
Fe	6.276175000	4.003771000	6.900639000
S	5.070442000	5.483914000	8.219423000
S	7.591399000	4.370690000	10.657998000
S	8.437942000	4.715304000	7.031661000
S	6.250293000	2.205375000	8.172368000
Cl	9.419844000	1.181933000	8.954034000
Cl	3.985352000	3.295297000	11.130173000
Cl	5.509842000	3.923833000	4.805324000
C	7.814057000	7.909048000	10.760795000
C	9.119846000	7.550493000	10.312765000
C	7.680880000	8.836790000	11.805769000
O	9.183118000	6.689525000	9.305102000
Cl	6.074784000	9.260415000	12.321506000
C	10.245830000	8.108442000	10.931732000
C	8.818420000	9.399621000	12.419162000
Cl	11.814736000	7.613406000	10.366312000
Cl	8.626053000	10.537559000	13.716855000
C	10.102016000	9.033225000	11.985573000
Cl	11.511337000	9.705577000	12.744809000
O	6.793773000	7.338402000	10.142246000
C	7.232887000	9.624927000	5.886628000
H	8.101529000	10.256617000	6.117637000
H	6.311424000	10.196045000	6.064441000
H	7.274578000	9.321542000	4.831601000
C	7.248537000	8.453076000	6.730170000
N	7.260296000	7.510452000	7.402761000
H	11.068127000	3.790251000	8.580187000
N	10.390379000	4.034459000	9.308162000
H	10.325408000	5.064879000	9.323588000
H	10.812170000	2.602371000	10.599080000
N	10.980735000	3.618783000	10.580976000
H	10.332232000	3.984061000	11.290004000

[Mo]-N_2H_4 – Fe3 Terminal

Mo	5.565906000	5.409006000	7.645987000
Fe	4.502754000	2.875530000	7.938609000
Fe	7.347232000	3.471336000	8.328276000
Fe	6.154405000	3.463982000	5.663173000
S	4.077523000	4.271192000	6.226859000
S	5.531294000	4.101957000	9.594583000
S	7.666701000	4.956790000	6.712728000
S	6.400392000	1.720468000	7.118276000
Cl	9.169370000	2.976806000	9.540074000
Cl	2.778199000	1.606108000	8.577899000
Cl	7.043383000	4.151676000	3.567588000
C	4.217577000	7.550274000	9.100646000
C	5.606667000	7.813582000	9.293988000
C	3.267649000	8.390508000	9.700908000
O	6.451263000	6.990429000	8.695252000
Cl	1.583721000	8.040584000	9.436713000
C	6.000027000	8.905903000	10.081001000
C	3.671963000	9.485285000	10.491870000
Cl	7.703380000	9.193661000	10.288377000
Cl	2.476720000	10.508173000	11.228357000
C	5.038203000	9.743136000	10.682178000
Cl	5.549270000	11.087528000	11.656161000
O	3.914693000	6.510034000	8.343585000
C	5.396165000	8.838086000	4.249471000
H	6.223176000	9.545217000	4.401423000
H	4.441263000	9.377219000	4.312890000
H	5.489311000	8.379324000	3.255687000
C	5.443301000	7.809938000	5.262947000
N	5.484512000	6.980013000	6.069007000
H	4.705354000	2.595135000	3.533832000
N	5.117419000	2.050032000	4.296831000
H	5.837033000	1.471160000	3.852644000
H	4.491991000	0.725415000	5.631515000
N	4.084595000	1.137288000	4.778943000
H	3.345322000	1.751047000	5.144528000

[V]$^{3+}$-N_2H_4 – Fe1 Terminal

V	3.210390000	5.099683000	7.657896000
Fe	2.633634000	2.468882000	7.712858000
Fe	4.808588000	3.578384000	9.351777000
Fe	5.101902000	3.280415000	6.522648000
S	2.988668000	3.660813000	5.853386000
S	2.579661000	3.842505000	9.508406000
S	5.525817000	5.108490000	7.853162000
S	4.768661000	1.678327000	8.094801000
Cl	5.944552000	3.592780000	11.282969000
Cl	2.015272000	0.333012000	7.000842000
Cl	6.546986000	2.987184000	4.858845000
C	0.810149000	6.536228000	8.207423000
C	1.824670000	7.198730000	8.969104000
C	-0.529683000	6.917082000	8.356985000
O	3.057611000	6.767831000	8.806093000
Cl	-1.730482000	6.048561000	7.439368000
C	1.452104000	8.247332000	9.827215000
C	-0.891787000	7.964753000	9.229790000
Cl	2.708829000	9.048470000	10.729758000
Cl	-2.562204000	8.416185000	9.397957000
C	0.101449000	8.632472000	9.960005000
Cl	-0.327123000	9.929133000	11.036637000
O	1.228329000	5.579873000	7.395317000
C	3.730602000	8.459458000	4.320934000
H	3.993014000	9.412739000	4.800253000
H	2.780215000	8.575987000	3.781767000
H	4.519140000	8.185571000	3.606378000
C	3.599723000	7.425798000	5.324143000
N	3.497659000	6.593605000	6.123962000
N	0.643215000	2.835199000	7.572372000
H	0.320531000	2.453097000	6.677410000
H	0.535071000	3.870594000	7.541957000
H	-0.024721000	1.291561000	8.615818000
N	-0.221351000	2.300474000	8.594120000
H	0.137463000	2.676838000	9.480587000

[V]$^{3+}$-N_2H_4 – Fe2 Terminal

V	7.367222000	5.992546000	8.808981000
Fe	5.679052000	3.961416000	9.740227000
Fe	8.412733000	3.486161000	8.826906000
Fe	6.389665000	3.860972000	6.974600000
S	5.199136000	5.465825000	8.114873000
S	7.637058000	4.585754000	10.659500000
S	8.391087000	4.817230000	7.024432000
S	6.509614000	2.231306000	8.514509000
Cl	9.671778000	1.585823000	8.255930000
Cl	3.991474000	3.521186000	11.164894000
Cl	5.496146000	3.275408000	5.019194000
C	7.841413000	7.931653000	10.771439000
C	9.152968000	7.566033000	10.331391000
C	7.700750000	8.766761000	11.890070000
O	9.204706000	6.804898000	9.248854000
Cl	6.091087000	9.195042000	12.392804000
C	10.272035000	8.026848000	11.036882000
C	8.834442000	9.231143000	12.588472000
Cl	11.845430000	7.527659000	10.484741000
Cl	8.633800000	10.254639000	13.977698000
C	10.119617000	8.858950000	12.165388000
Cl	11.522390000	9.411032000	13.027833000
O	6.841025000	7.456285000	10.055804000
C	7.166659000	9.539161000	5.659364000
H	8.184718000	9.812923000	5.350350000
H	6.680908000	10.411987000	6.116433000
H	6.592773000	9.227347000	4.775928000
C	7.220339000	8.453202000	6.612200000
N	7.260706000	7.576108000	7.367908000
H	10.883553000	3.981966000	8.596708000
N	10.232616000	4.202546000	9.356769000
H	10.145616000	5.237724000	9.421874000
H	10.825837000	2.680660000	10.472751000
N	10.823675000	3.704088000	10.573535000
H	10.139334000	3.910117000	11.312370000

[V]$^{3+}$-N_2H_4 – Fe3 Terminal

V	5.517844000	5.516799000	7.761778000
Fe	4.618813000	2.786470000	7.969841000
Fe	7.325891000	3.429388000	8.340918000
Fe	6.084873000	3.695732000	5.648127000
S	4.045355000	4.330671000	6.438375000
S	5.558966000	4.103596000	9.581816000
S	7.579786000	5.027656000	6.847670000
S	6.466153000	1.842555000	6.976931000
Cl	8.995128000	2.715457000	9.628402000
Cl	3.092277000	1.403332000	8.781293000
Cl	6.826925000	4.641477000	3.611719000
C	4.182664000	7.568350000	9.198822000
C	5.574432000	7.831626000	9.396562000
C	3.232601000	8.413945000	9.792129000
O	6.403650000	6.993954000	8.808292000
Cl	1.546402000	8.064525000	9.526336000
C	5.965014000	8.926434000	10.181827000
C	3.635944000	9.513938000	10.578660000
Cl	7.669786000	9.211571000	10.400221000
Cl	2.438365000	10.545600000	11.302349000

C	5.001135000	9.770215000	10.773778000
Cl	5.510672000	11.121994000	11.740819000
O	3.897863000	6.519850000	8.456168000
C	5.560940000	8.357080000	3.942314000
H	6.381494000	9.086265000	3.984079000
H	4.616241000	8.885080000	3.755062000
H	5.751335000	7.644841000	3.126826000
C	5.486424000	7.629507000	5.188351000
N	5.440625000	7.017938000	6.171528000
H	4.611218000	2.957356000	3.538371000
N	5.051751000	2.355240000	4.240632000
H	5.759759000	1.820511000	3.727187000
H	4.519453000	0.803673000	5.362717000
N	4.039233000	1.398301000	4.674855000
H	3.388721000	1.952895000	5.248052000

[V]$^{2+}$-N_2H_4 – Fe1 Terminal

V	3.225785000	5.090441000	7.626835000
Fe	2.700877000	2.413952000	7.693088000
Fe	4.861249000	3.555029000	9.284116000
Fe	5.138581000	3.337404000	6.464093000
S	2.989501000	3.646664000	5.804756000
S	2.609909000	3.835255000	9.505707000
S	5.546468000	5.152745000	7.839263000
S	5.029044000	1.674373000	7.934285000
Cl	6.011080000	3.449829000	11.258352000
Cl	1.947068000	0.113767000	7.859974000
Cl	6.607805000	3.241991000	4.729371000
C	0.814730000	6.523261000	8.197553000
C	1.827938000	7.184713000	8.973986000
C	-0.527044000	6.900189000	8.353468000
O	3.061908000	6.783473000	8.796660000
Cl	-1.722087000	6.052277000	7.404085000
C	1.438236000	8.208817000	9.855827000
C	-0.901313000	7.927397000	9.247426000
Cl	2.688877000	9.002794000	10.780285000
Cl	-2.577839000	8.364366000	9.418458000
C	0.083860000	8.583287000	9.996330000
Cl	-0.356557000	9.851640000	11.105310000
O	1.231598000	5.588219000	7.371441000
C	3.765801000	8.476799000	4.324852000
H	3.967979000	9.436623000	4.819463000
H	2.845365000	8.565267000	3.731700000
H	4.602399000	8.233741000	3.655439000
C	3.613477000	7.433479000	5.316401000
N	3.499645000	6.591359000	6.104801000
N	0.532189000	2.790604000	7.446196000
H	0.138353000	2.377091000	6.596265000
H	0.470142000	3.818667000	7.344124000
H	-0.054395000	1.442239000	8.758392000
N	-0.353793000	2.406870000	8.549647000
H	0.019719000	2.943425000	9.344991000

[V]$^{2+}$-N_2H_4 – Fe2 Terminal

V	7.354311000	5.848487000	8.806002000
Fe	5.631039000	3.825625000	9.666237000
Fe	8.359628000	3.313049000	8.787548000
Fe	6.287867000	4.017531000	6.909392000
S	5.118118000	5.504576000	8.239341000
S	7.597539000	4.428138000	10.653376000
S	8.426882000	4.763795000	7.036270000
S	6.274628000	2.180130000	8.158222000
Cl	9.465406000	1.165317000	9.030752000
Cl	3.973912000	3.219187000	11.119171000
Cl	5.481565000	3.966575000	4.782615000
C	7.813562000	7.893128000	10.743129000
C	9.128649000	7.533123000	10.287690000
C	7.695330000	8.807905000	11.804928000
O	9.185332000	6.692152000	9.276895000
Cl	6.086661000	9.235190000	12.332101000
C	10.250950000	8.082272000	10.924849000
C	8.834385000	9.360248000	12.430286000
Cl	11.827176000	7.593210000	10.354895000
Cl	8.643644000	10.484295000	13.746653000
C	10.113983000	8.995621000	11.993351000
Cl	11.530321000	9.653556000	12.762843000
O	6.802305000	7.337605000	10.122834000
C	7.214948000	9.600401000	5.881453000
H	8.074216000	10.249555000	6.098563000
H	6.287335000	10.165813000	6.045306000
H	7.261140000	9.281880000	4.830996000
C	7.244856000	8.441483000	6.748075000
N	7.268464000	7.508948000	7.436228000
H	11.083558000	3.864679000	8.588682000
N	10.377917000	4.072261000	9.301135000
H	10.255321000	5.099994000	9.320887000
H	10.785970000	2.657078000	10.608702000
N	10.953082000	3.674855000	10.590165000
H	10.271361000	4.034738000	11.272043000

[V]$^{2+}$-N_2H_4 – Fe3 Terminal

V	5.497409000	5.516148000	7.719382000
Fe	4.442470000	2.968834000	8.068371000
Fe	7.223971000	3.513561000	8.387032000
Fe	6.028108000	3.420900000	5.677491000
S	4.010382000	4.363119000	6.351868000
S	5.471755000	4.250289000	9.673882000
S	7.524951000	4.983653000	6.739714000
S	6.266902000	1.723475000	7.220100000
Cl	9.107476000	2.966063000	9.574127000
Cl	2.675611000	1.687478000	8.741236000
Cl	6.998232000	3.819530000	3.517546000
C	4.210652000	7.635876000	9.154441000
C	5.617973000	7.881572000	9.324452000
C	3.291064000	8.503896000	9.767819000
O	6.431332000	7.044158000	8.727382000
Cl	1.589990000	8.180582000	9.536255000
C	6.029243000	8.978506000	10.099685000
C	3.721388000	9.602938000	10.543567000
Cl	7.745509000	9.246040000	10.284060000
Cl	2.549255000	10.652640000	11.290240000
C	5.091263000	9.840627000	10.709880000
Cl	5.641344000	11.189096000	11.664934000
O	3.884917000	6.599279000	8.422946000
C	5.323081000	8.954776000	4.415961000
H	6.114732000	9.687487000	4.624221000
H	4.348212000	9.459841000	4.450700000
H	5.476076000	8.536444000	3.411731000
C	5.365365000	7.894793000	5.400535000
N	5.406436000	7.040274000	6.182437000
H	4.299587000	2.462794000	3.630930000
N	4.753573000	1.938946000	4.384617000
H	5.372057000	1.269991000	3.916640000
H	4.201096000	0.756588000	5.865793000
N	3.708363000	1.157029000	5.052479000
H	3.119788000	1.875272000	5.498520000

Cobaltocene

Co	6.344902000	-1.560863000	5.954199000
C	6.026534000	-0.546254000	7.802213000
C	5.793764000	-1.965780000	7.908293000
C	7.027387000	-2.657672000	7.660031000

C	7.975833000	-1.681438000	7.254384000
C	7.359991000	-0.371507000	7.359666000
H	5.287377000	0.234988000	7.955722000
H	4.853729000	-2.430438000	8.196202000
H	7.174910000	-3.733692000	7.679930000
H	7.845030000	0.573784000	7.130536000
C	7.003487000	-1.909758000	4.002425000
C	6.025606000	-2.892655000	4.310254000
C	4.831382000	-2.191450000	4.690131000
C	5.029806000	-0.780013000	4.468634000
C	6.380632000	-0.601438000	4.083778000
H	8.038286000	-2.097574000	3.727392000
H	6.179204000	-3.967252000	4.351089000
H	3.905520000	-2.652856000	5.025300000
H	6.875432000	0.346055000	3.886922000
H	4.297659000	0.002999000	4.644881000
H	8.999261000	-1.871726000	6.940971000

Cobaltocene+

Co	6.345484000	-1.559857000	5.953922000
C	5.993737000	-0.566517000	7.703084000
C	5.768980000	-1.972219000	7.868925000
C	6.988156000	-2.656083000	7.552446000
C	7.966330000	-1.672850000	7.190908000
C	7.351669000	-0.381399000	7.283979000
H	5.252195000	0.216883000	7.827700000
H	4.827707000	-2.439839000	8.142050000
H	7.132239000	-3.732332000	7.543410000
H	7.818983000	0.566878000	7.035793000
C	7.029624000	-1.894165000	4.059336000
C	6.050075000	-2.875978000	4.420774000
C	4.831650000	-2.190684000	4.736965000
C	5.058197000	-0.785217000	4.570822000
C	6.416566000	-0.601926000	4.151933000
H	8.066673000	-2.091617000	3.805039000
H	6.215439000	-3.947127000	4.487976000
H	3.912470000	-2.651902000	5.085650000
H	6.908160000	0.351000000	3.980371000
H	4.340517000	0.004461000	4.771737000
H	8.980884000	-1.874055000	6.860133000

Acetonitrile

C	4.650980000	2.415773000	0.401791000
H	5.188537000	1.460507000	0.472254000
H	5.375474000	3.239704000	0.453426000
H	4.124157000	2.460767000	-0.560904000
C	3.698826000	2.527098000	1.490976000
N	2.935392000	2.616351000	2.364266000

Hydrazine

N	11.308561000	13.026444000	8.304384000
H	11.089601000	13.969916000	8.644970000
H	10.558821000	12.423191000	8.650714000
N	11.234584000	12.983454000	6.853669000
H	12.131873000	13.331373000	6.507546000
H	10.527096000	13.644896000	6.513118000

Lutidinium Acid (Protonated)

C	11.785140000	0.431853000	-1.899193000
C	11.798729000	1.641927000	-1.204289000
C	11.719041000	1.638538000	0.183983000
N	11.629226000	0.432397000	0.803641000
C	11.611105000	-0.774046000	0.178285000
C	11.691181000	-0.777933000	-1.209945000
H	11.847836000	0.431616000	-2.987522000
H	11.870645000	2.593138000	-1.727689000
H	11.678736000	-1.729301000	-1.737901000
C	11.505743000	-1.991689000	1.030061000
H	11.512765000	-2.892469000	0.408423000
H	12.346084000	-2.038471000	1.738516000
H	10.575060000	-1.969284000	1.616544000
C	11.729223000	2.856333000	1.042008000
H	12.615421000	2.855463000	1.694511000
H	11.746731000	3.758228000	0.422160000
H	10.839169000	2.880803000	1.687747000
H	11.572692000	0.432517000	1.824982000

Lutidinium Acid (Deprotonated)

C	11.844251000	0.427942000	-1.904249000
C	11.834504000	1.629712000	-1.197037000
C	11.699742000	1.594531000	0.198174000
N	11.579540000	0.433404000	0.874418000
C	11.588678000	-0.730456000	0.192252000
C	11.719566000	-0.771035000	-1.203159000
H	11.947766000	0.425679000	-2.990685000
H	11.929688000	2.585882000	-1.712770000
H	11.722892000	-1.729116000	-1.724145000
C	11.452332000	-1.988594000	1.005804000
H	11.453125000	-2.881324000	0.367961000
H	12.280055000	-2.070126000	1.726365000
H	10.517923000	-1.970093000	1.586358000
C	11.682316000	2.855725000	1.018178000
H	12.506322000	2.849903000	1.747435000
H	11.779072000	3.747190000	0.385985000
H	10.744701000	2.928499000	1.589385000

Ammonia

N	-0.212595000	19.484250000	-5.102840000
H	-0.212638000	20.426191000	-4.700975000
H	0.603144000	19.013249000	-4.700976000
H	-1.028332000	19.013297000	-4.700971000
